# Eddy Current Measurement for Planar Structures

**DOI:** 10.3390/s22228695

**Published:** 2022-11-10

**Authors:** Zihan Xia, Ruochen Huang, Ziqi Chen, Kuohai Yu, Zhijie Zhang, Jorge Ricardo Salas-Avila, Wuliang Yin

**Affiliations:** 1 School of Electrical and Electronic Engineering, University of Manchester, Manchester M13 9PL, UK; 2 College of Electrical Engineering and Automation, Fuzhou University, Fuzhou 350108, China; 3 School of Instrument and Electronics, North University of China, Taiyuan 030051, China; 4 MAIERIC Ltd., Manchester M15 6SZ, UK

**Keywords:** eddy current testing, electromagnetic induction, planar structure, theoretical calculation, measurement

## Abstract

Eddy current (EC) testing has become one of the most common techniques for measuring metallic planar structures in various industrial scenarios such as infrastructures, automotive, manufacturing, and chemical engineering. There has been significant progress in measuring the geometry, electromagnetic properties, and defects of metallic planar structures based on electromagnetic principles. In this review, we summarize recent developments in EC computational models, systems, algorithms, and measurement approaches for planar structures. First, the computational models including analytical models, numerical methods, and plate property estimation algorithms are introduced. Subsequently, the impedance measurement system and probes are presented. In plate measurements, sensor signals are sensitive to probe lift-off, and various algorithms for reducing the lift-off effect are reviewed. These approaches can be used for measureing thickness and electromagnetic properties. Furthermore, defect detection for metallic plates is also discussed.

## 1. Introduction

Measuring the defect and physical properties (electromagnetic properties, dimensions etc.) of a planar structure is important in a range of technological applications, for example, coating surface treatments [1] and quality inspections [2,3]. Inspections during the manufacturing process and on-site monitoring of steel plates is key to the safety and efficiency of industrial applications in railway, aircraft, and nuclear facilities [4,5,6,7,8,9]. Furthermore, measuring nano-scale copper film thickness guarantees product quality of the integrated circuit manufacturing process in the semiconductor industry [10,11] and chemical mechanical planarization process [12]. As one of the common nondestructive testing (NDT) techniques, for decades, EC testing has been intensively investigated for measuring planar structures, due to its characteristics of being noninvasive, contactless, fast response, and relatively low cost. The technique employs a time-varying magnetic field excited by coils covering the region of interest (ROI) in conductive structures. The eddy current is induced in the measured structures by an excitation magnetic field. Variations in the magnetic field, caused by the eddy current, contain the physical properties and geometric information of the ROI, which can be detected and employed for structural analysis.

Forward and inverse problems are the primary theoretical issues of EC testing. The forward problem aims to calculate the frequency-dependent measurements for given metallic objects, while the inverse problem determines the physical properties of the object from the frequency-dependent measurement. The forward problem can be solved by analytical and numerical methods. The analytical methods include the Dodd and Deeds model [13,14] for symmetric geometry, and the second-order vector potential (SOVP) method, also known as the Hertz method, for asymmetric geometry [15]. The numerical methods cover the finite difference method, finite element method (FEM), and boundary element method (BEM). Recent studies on analytical models for plate measurement have focused on the solution to complex structures, for example, finite-size plates [16] and model simplification that facilitates the direct estimation of plate properties [17]. The FEM method has been implemented for complex geometry and anisotropic materials [18,19,20,21]. In addition, 3D models of FEM have been accelerated for defect plates [22,23]. In the inverse problem, the plate properties can be estimated according to the linearized simplified analytical model with lift-off compensation strategy [24] and multivariable optimization algorithms [25,26]. These methods deepen the understanding of the phenomenon in EC testing and account for the relationships between the measurement signals and plate properties.

The implementation of EC testing consists of a data acquisition system and probe which are used to obtain the measurement signal. The customized and commercial impedance analyzers both enable the measurement of mutual inductance between coils and other magnetic field variables. In general, EC probes designed for plate property estimation and defect scanning contain transmitting (Tx) coils and receiving (Rx) elements. According to the required EM field for specific applications, the structure and dimensions of Tx coils can be selected and optimized. Furthermore, coils or magnetic field sensors are usually chosen as the Rx elements, depending on the induced EM field by test pieces. 

As compared with previous review articles on eddy current testing [27,28,29,30], this work investigates recent improvements of multi-frequency EC testing in plate measurement in terms of a computational model and measurement system. In addition, the key issues affecting measurement signals, i.e., lift-off and tilt effect of the probe, are discussed theoretically.

The arrangement of the rest of the paper is as follows: In Section 2, we describe the computational methods of EC testing, including the solution of the forward problem by the analytical and FEM methods and the inverse problem by the optimization approach; in Section 3, we illustrate the recently developed impedance measurement systems with relevant calibration strategies and characteristics of EC probes for plate measurement; in Section 4, we explain the lift-off compensation methods and defect scanning applications.

## 2. Computational Models

### 2.1. Analytical Models

#### 2.1.1. Dodd and Deeds and Associated Simplified Models

**Figure 1:**
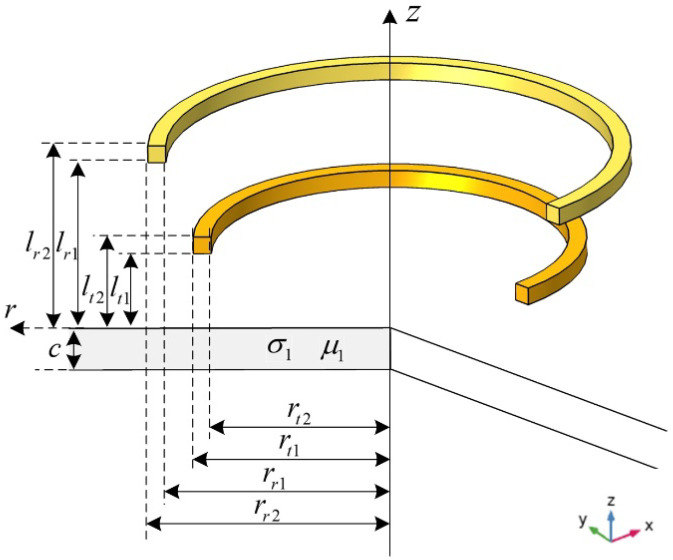
Metallic plate measured using a probe with transmitting and receiving coils.

For the single-layered plate model placed near a probe consisting of Tx and Rx coils, as shown in Figure 1, according to the Dodd and Deeds model [13,14], the complex mutual inductance [31], referred to as inductance hereinafter, between the Tx and the Rx coils, caused by the metallic plate can be calculated by:

(1) $$\Delta L\left(\omega \right)=\frac{\Delta Z}{j\omega }\;\begin{array}{c} =\frac{\pi N_{t}N_{r}\mu_{0}}{\left(l_{t2}-l_{t1}\right)\left(l_{r2}-l_{r1}\right)\left(r_{t2}-r_{t1}\right)\left(r_{r2}-r_{r1}\right)}\int_{0}^{\infty }\frac{J\left(r_{t2},r_{t1}\right)J\left(r_{r2},r_{r1}\right)}{\alpha^{6}}\left(e^{-\alpha l_{t1}}-e^{-\alpha l_{t2}}\right)\left(e^{-\alpha l_{r1}}-e^{-\alpha l_{r2}}\right)\phi \left(\alpha \right)d\alpha \end{array}$$

(2) $$\phi \left(\alpha \right)=\frac{\left(\alpha_{1}+\mu_{1}\alpha \right)\left(\alpha_{1}-\mu_{1}\alpha \right)-\left(\alpha_{1}+\mu_{1}\alpha \right)\left(\alpha_{1}-\mu_{1}\alpha \right)e^{2\alpha_{1}c}}{-\left(\alpha_{1}-\mu_{1}\alpha \right)\left(\alpha_{1}-\mu_{1}\alpha \right)+\left(\alpha_{1}+\mu_{1}\alpha \right)\left(\alpha_{1}+\mu_{1}\alpha \right)e^{2\alpha_{1}c}}$$

(3) $$J\left(r_{t2},r_{t1}\right)=\int_{\alpha r_{t1}}^{\alpha r_{t2}}\tau J_{1}\left(\tau \right)\tau$$

where ω is the angular frequency of excitation; α denotes the spatial frequency; $\alpha_{1}=\sqrt{\alpha^{2}+j\omega \mu_{0}\mu_{1}\sigma_{1}}$; $\sigma_{1}$ and $\mu_{1}$ are electrical conductivity and relative magnetic permeability of plate, respectively, $J_{1}\left(\cdot \right)$ is the Bessel function of the first kind; $N_{t}$ and $N_{r}$ are the number of turns for the Tx and Rx coils, respectively.

The inductance can be calculated through numerical integration, whereas it is time-consuming. The truncated region eigenfunction expansion (TREE) method, proposed by T. Theodoulidis and E. Kriezis, recasts the expression as sums rather than integrals [15,32], i.e.:

(4) $$\Delta L\left(\omega \right)=\frac{2\pi N_{t}N_{r}\mu_{0}}{\left(l_{t2}-l_{t1}\right)\left(l_{r2}-l_{r1}\right)\left(r_{t2}-r_{t1}\right)\left(r_{r2}-r_{r1}\right)}\underset{\alpha_{i}}{\sum }\frac{J\left(r_{t2},r_{t1}\right)J\left(r_{r2},r_{r1}\right)}{{\left(h\left(J_{0}\left(\alpha_{i}h\right)\right)\right)}^{2}\alpha_{i}{}^{5}}\left(e^{-\alpha_{i}l_{t1}}-e^{-\alpha_{i}l_{t2}}\right)\left(e^{-\alpha_{i}l_{r1}}-e^{-\alpha_{i}l_{r2}}\right)\phi \left(\alpha_{i}\right)$$

where $\;\alpha_{i}$ satisfies the condition on the truncated boundary, $J_{1}\left(\alpha_{i}h\right)=0$, $i\in \left\{1,2,\ldots ,n_{\alpha }\right\}$, which indicates that the electromagnetic field vanishes at the boundary r=h. The series form accelerates the inductance calculation which guarantees the accuracy if the range of spatial frequencies is appropriate.

**Figure 2:**
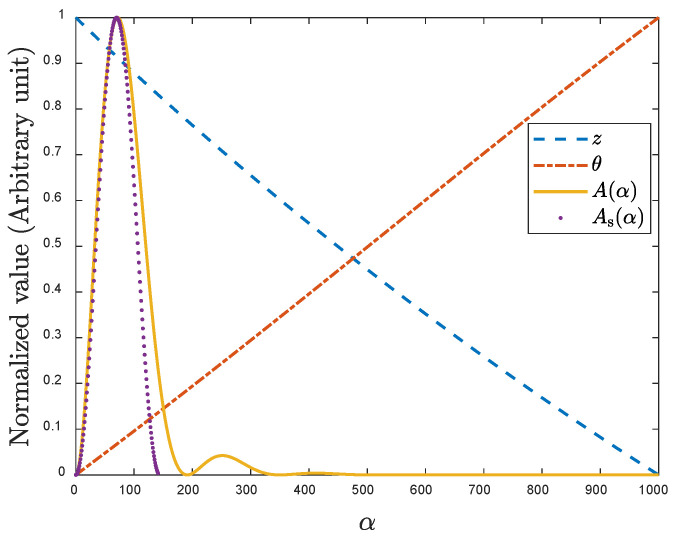
Comparison between the plate term $\phi \left(\alpha \right)=ze^{i\theta }$ and sensor term A(α) together with its approximation function $A_{s}\left(\alpha \right)$.

Since the plate term ϕ(α), sometimes referred to as phase term, varies relatively slowly as compared with the sensor term, as the representative curves shown in Figure 2, W. Yin et al. employed the dominant value of plate term to approximate the original one [33], i.e.:

(5) $$\Delta L\left(\omega \right)=\phi \left(\alpha_{0}\right)\Delta L_{0}$$

(6) $$\Delta L_{0}=\int_{0}^{\infty }A\left(\alpha \right)d\alpha$$

(7) $$A\left(\alpha \right)=\frac{\pi N_{t}N_{r}\mu_{0}}{\left(l_{t2}-l_{t1}\right)\left(l_{r2}-l_{r1}\right)\left(r_{t2}-r_{t1}\right)\left(r_{r2}-r_{r1}\right)}\frac{J\left(r_{t2},r_{t1}\right)J\left(r_{r2},r_{r1}\right)}{\alpha^{6}}\left(e^{-\alpha l_{t1}}-e^{-\alpha l_{t2}}\right)\left(e^{-\alpha l_{r1}}-e^{-\alpha l_{r2}}\right)$$

where $\alpha_{0}$ is the characteristic/dominant spatial frequency corresponding to the maximum value of the sensor term A(α) which is determined by the sensor structure including the sensor lift-off.

If the sensor structure is known or can be inferred, one can obtain $\Delta L_{0}$ and $\alpha_{0}$ before measurement. The approximation accelerates the inductance calculation and facilitates the estimation of plate properties using the plate term $\phi \left(\alpha_{0}\right)$. In addition, the sensor term A(α) which reaches its maximum value when $\alpha =\alpha_{0}$ can be approximated by an elementary function $A_{s}\left(\alpha \right)$:

(8) $$A_{s}\left(\alpha \right)=A_{m}\left(\alpha_{0}\right)e^{-2\alpha l_{0}}\sin^{2}\left(\frac{\alpha \pi }{2\alpha_{0}}\right)$$

where $A_{m}\left(\alpha_{0}\right)$ is the maximum value of sensor term without the lift-off.

#### 2.1.2. Model for the Finite-Size Plate

In the theoretical analysis, the Dodd and Deeds model in Equations (1)–(3) calculates the inductance of plates with infinite radius, whereas in practical measurements, the plates normally have finite radiuses.

**Figure 3:**
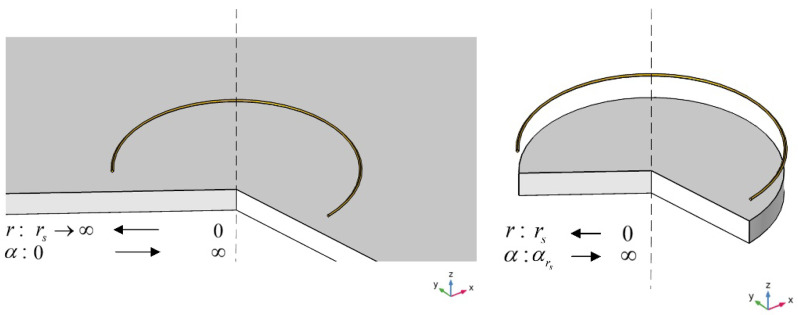
Relationship between the spatial frequency and radius, for the measurement of a finite-size plate.

The TREE method indicates that the EC of the finite-size geometry can be evaluated by considering a certain range of spatial frequencies in the analytical model. By analyzing the analytical solution, R. Huang et al. proposed that the spatial frequency α was inversely proportional to the plate radius [16,34,35], as the relationship shown in Figure 3. This means that the limits of integration for α relates to the plate radius. For the plate with a radius of $r_{s}$, the range is $\alpha \in \left[\alpha_{r_{s}},\infty \right]$, $\alpha_{r_{s}}=x_{0}/r_{s}$, and $x_{0}=3.518$. Consequently, the inductance change caused by the presence of the plate is:

(9) $$\Delta L\left(\omega \right)=\int_{\alpha_{r_{s}}}^{\infty }A\left(\alpha \right)\phi \left(\alpha \right)d\alpha$$

Then, based on the TREE method, the finite region eigenfunction expansion (FREE) method is proposed which confines the terms of series relating to the plate radius, i.e.:

(10) $$\Delta L\left(\omega \right)=\overset{\alpha =\alpha_{n_{\alpha }}}{\underset{\alpha =\alpha_{r_{s}}}{\sum }}A\left(\alpha \right)\phi \left(\alpha \right)d\alpha$$

### 2.2. Finite Element Method

The finite element method (FEM) is a universal computation tool for arbitrary sensor setup and the geometry of a target object. With the support of Galerkin equations [36], the unknown fields are subjected to the boundary conditions, and the approximated vector and scalar potentials are:

(11) $$\int_{\Omega_{c}}\nabla \times N_{i}\cdot v\nabla \times A^{n}d\Omega +\int_{\Omega_{c}}j\omega \sigma N_{i}\cdot A^{n}d\Omega +\int_{\Omega_{c}}j\omega \sigma N_{i}\cdot \nabla V^{n}d\Omega =\int_{\Omega_{c}}\nabla \times N_{i}\cdot v_{0}\nabla \times A_{s}\begin{array}{cc} d\Omega & i \end{array}=1,2,\ldots ,6$$

(12) $$\int_{\Omega_{c}}j\omega \sigma \nabla L_{i}\cdot A^{n}d\Omega +\int_{\Omega_{c}}j\omega \sigma \nabla L_{i}\cdot \nabla V^{n}d\Omega =0\;i=1,2,\ldots ,4$$

where $v_{0}$ is the reluctivity in the air, v is the reluctivity, ω is the excitation frequency, $A^{n}$ and $V^{n}\;$ are vector potential and scalar potential in element n, respectively.

As compared with analytical methods, the FEM method can adapt to any geometry with high accuracy. However, it has a high computation load and requires a large amount of time to obtain the final solution for a single simulation. An equivalent phenomenon was observed for a thin structure by using an analytical method. From the principle of a plane wave reflected and transmitted at the surface of a nonmagnetic plate, with a geometry of thin thickness, the inductance ΔL can be simplified as [37]:

(13) $$\Delta L=\frac{2\pi rE_{0}}{I}\frac{-\mu_{0}\sigma c}{\alpha_{0}+j\omega \mu_{0}\sigma c}$$

where r is the coil radius, $E_{0}$ is the electrical field in the air transmitted from the probe. 

Then, considering two plates in different materials and thicknesses, the induced inductance is approximately equal when:

(14) $$\frac{D_{1}}{D_{2}}=\frac{\sigma_{2}}{\sigma_{1}}$$

where $D_{1}$ and $D_{2}$ are the thicknesses of the plates, and $\sigma_{1}$ and $\sigma_{2}$ are the corresponding conductivity.

**Figure 4:**
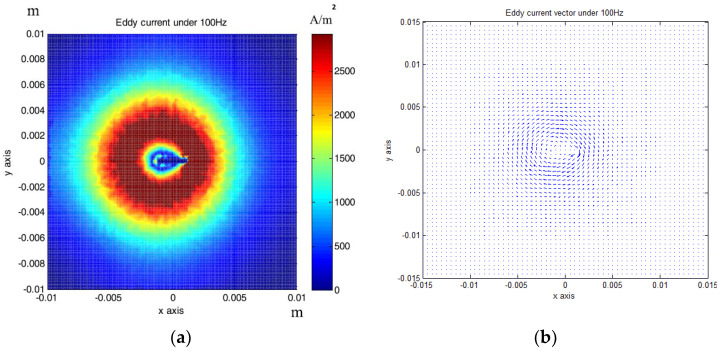
Eddy current performance using an accelerated FEM solver: (**a**) Color map; (**b**) quiver map [42].

The above relationship reduces the computational burden when modeling thin structure samples. Researchers have been devoted to speed up the computation speed to satisfy users’ requirements by using some approximations/techniques, i.e., zero-thickness defect [38], dedicated kernel [39], and the FEM-BEM hybrid method [40,41]. M. Lu and W. Yin et al. introduced a preconditioner in the computation for EC problems [42,43]. The EC performance using this accelerated method for a co-axial air-cored probe above a defect is shown in Figure 4.

**Figure 5:**
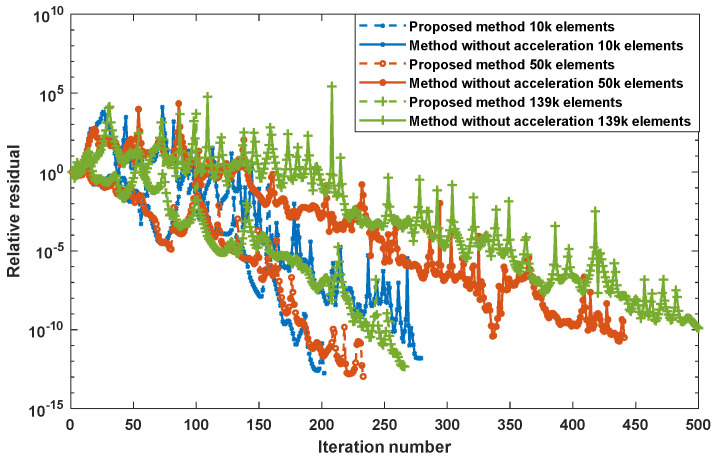
The relative residual with different mesh elements [22].

The proposed preconditioning method optimizes the initial guess for solving the system algebraic equations. Combined with the perturbed matrix inversion (PMI) method, a fast FEM approach has been proposed which can effectively shrink the time needed for the crack computation due to the small perturbation [23]. Furthermore, by exploiting the fact that the perturbed field exists in the surrounding region of the crack, the simulation solution can be calculated by combining the crack affected field and the original field without crack [22]. Table 1 lists the acceleration rate and computation deviation using the proposed method under different mesh elements. It can be noticed that, for the mesh including 139 k elements, the computation time employing the accelerated method can be decreased by 34 times. As can be seen from Figure 5, the iteration number is reduced, especially for large-scale mesh models.

### 2.3. Tilted Structure Measurement

The probe tilt is identified as one of the major sources of noise in EC surface inspections [44]. The FEM and BEM methods can model the tilted probes naturally [45], while, for the analytical model, the geometry is no longer axisymmetric and special treatments are required to represent the electromagnetic field [15]. The tilt effect has been studied for decades employing the methods including the SOVP [46], and the electromagnetic field of arbitrary current source above a conductive half-space [47]. The latter approach is analyzed as described below.

Following studies [44,48], applying the 2D Fourier transform and according to the Parseval theorem, the general expression of mutual inductance change due to the conductive half-space is:

(15) $$\begin{array}{c} \Delta L=2\mu_{0}\int_{0}^{\infty }\int_{0}^{\infty }\frac{1}{\kappa }\;{\tilde{h}}_{T}\left(u,v\right){\tilde{h}}_{R}\left(-u,-v\right)\frac{\mu_{r}\kappa -\kappa_{1}}{\mu_{r}\kappa +\kappa_{1}}dudv\\ {\tilde{h}}_{T}\left(u,v\right)=\frac{N_{t}}{2\left(r_{t2}-r_{t1}\right)\left(l_{t2}-l_{t1}\right)\kappa^{3}}J\left(\kappa r_{t1},\kappa r_{t2}\right)\left(e^{-\kappa l_{t2}}-e^{-\kappa l_{t1}}\right)\\ {\tilde{h}}_{T}\left(u,v\right)=\frac{N_{r}}{2\left(r_{r2}-r_{r1}\right)\left(l_{r2}-l_{r1}\right)\kappa^{3}}J\left(\kappa r_{r1},\kappa r_{r2}\right)\left(e^{-\kappa l_{r2}}-e^{-\kappa l_{r1}}\right) \end{array}$$

where the source terms ${\tilde{h}}_{T}\left(u,v\right)$ and ${\tilde{h}}_{R}\left(u,v\right)$ indicate the 2D Fourier transformed magnetic field intensity on surface of plane induced by the Tx and Rx coils, respectively, $\kappa =\sqrt{u^{2}+v^{2}}$ and $\kappa_{1}=\sqrt{\kappa^{2}+j\omega \mu_{0}\mu_{r}\sigma }$.

**Figure 6:**
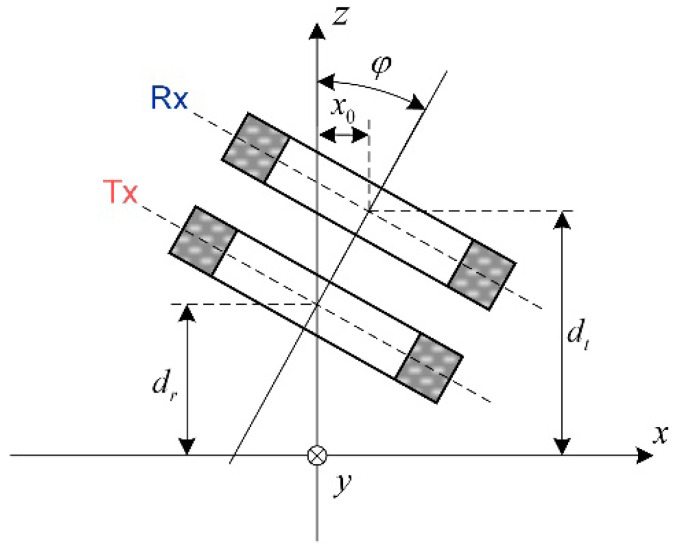
Cross-sectional view of tilted Tx-Rx coils, other dimensions similar as the counterparts without tilt angle.

As the 2D Fourier transform can characterize the rotation and translation function, for the probe shown in Figure 6 which is rotated around the y axis, the source terms become:

(16) $$\begin{array}{c} {\tilde{h}}_{T}^{’}\left(u,v\right)=\frac{jN_{t}}{2\left(r_{t2}-r_{t1}\right)\left(l_{t2}-l_{t1}\right)\psi^{3}}M\left(\psi r_{t1},\psi r_{t2}\right)e^{-\kappa d_{t}}\sin\left(\frac{\psi \left(l_{t2}-l_{t1}\right)}{2}\right)\\ {\tilde{h}}_{R}^{’}\left(u,v\right)=\frac{jN_{r}}{2\left(r_{r2}-r_{r1}\right)\left(l_{r2}-l_{r1}\right)\psi^{3}}M\left(\psi r_{r1},\psi r_{r2}\right)e^{-\kappa d_{r}}\sin\left(\frac{\psi \left(l_{r2}-l_{r1}\right)}{2}\right)\\ M\left(\psi r_{t1},\psi r_{t2}\right)=\int_{\psi r_{t1}}^{\psi r_{t2}}\tau I_{1}\left(\tau \right)d\tau \end{array}e^{jux_{0}}$$

where ψ=usinφ+jκcosφ.

Substituting the source terms in Equation (15) by ${\tilde{h}}_{T}^{’}\left(u,v\right)$ and ${\tilde{h}}_{R}^{’}\left(u,v\right)$, the inductance variation of the tilted probe can be calculated. The source terms in Equation (16) can be derived from the model of perpendicular coils [48].

For the measurement of a plate with an infinitely long crack, according to the thin-skin model in high-frequency regime [49,50], the inductance variation is:

(17) $$\begin{array}{c} \Delta L=\frac{-j\mu_{0}}{2\pi }\int_{-\infty }^{\infty }\frac{1}{v^{2}}{\tilde{H}}_{y,T}\left(v\right){\tilde{H}}_{y,R}\left(-v\right)\gamma dv\\ {\tilde{H}}_{y,T}\left(v\right)=jv\int_{-\infty }^{\infty }\frac{1}{\kappa \psi^{3}}M\left(\psi r_{t1},\psi r_{t2}\right)e^{-\kappa d_{t}}\sin\left(\frac{\psi \left(l_{t2}-l_{t1}\right)}{2}\right)\frac{\kappa_{1}}{\kappa_{1}+\kappa \mu_{r}}e^{jux_{0}}du \end{array}$$

where the term γ is determined by the crack parameters and plate properties, which are defined in [44,51].

The measurements of probe impedance scanning through the finite-size test pieces can reflect the electromagnetic properties of the plate under measurement [52]. However, the tilt effect makes it difficult to estimate the electromagnetic properties from the measurements. Through extensive experiments, it has been observed that at several specific excitation frequencies, for example, 40 kHz, the endpoint of inductance trajectory on the complex plane can characterize the conductivity of test pieces, and the phase of the point is almost invariant regarding the tilt angle ranging from $0^{o}$ to $16.7^{o}\;$ [53]. This phenomenon is shown in Table 2. In addition, the approximate linear relationship between the endpoint inductance phase and conductivity can be obtained through the curve fitting employing the least squares method. This facilitates the classification of nonmagnetic metals [54,55]. 

### 2.4. Multivariable Inversion

**Figure 7:**
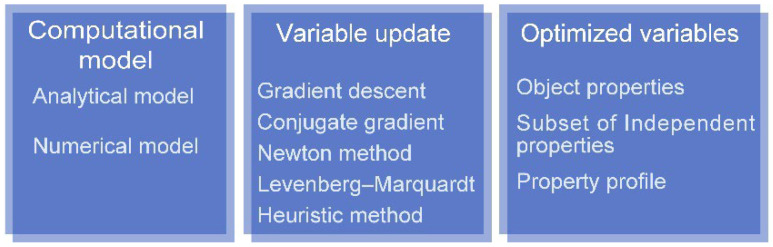
Primary issues of parameter inversion.

The inverse methods, closely related to the forward model, retrieve the plate properties and parameter distributions, for example, conductivity profile and defect shape from the measurements. Even though the forward model varies from different governing equations and object geometry, similar inverse methods have been widely employed for a variety of objects under measurement, including pipes and spheres. The notable implementations of inverse methods in EC testing are summarized in Figure 7. The prevalent inversion algorithms for parameter estimation can be found in the literature [25,56], without being exhaustive.

The inversion of physical properties is realized through optimization by minimizing the discrepancy between the measured and calculated electromagnetic measurements. A least square index is usually adopted as the objective function, which represents the Euclidean distance between the measured and calculated values. For instance, the objective function employing the coil inductance reads:

(18) $$\underset{v}{\min}f=\frac{1}{2}\|\Delta L\left(v\right)-\Delta L_{m}{\|}_{2}^{2}$$

where $v\in {\mathbb{R}}^{n_{v}\times 1}$ ($n_{v}$ denotes the quantity of variables), $\|\cdot {\|}_{2}^{2}$ represents the $\ell_{2}$-norm, ΔL(v) and $\Delta L_{m}\left(v\right)\in {\mathbb{C}}^{n_{f}\times 1}$ are calculated and measured inductance spectrum due to the test piece, respectively ($n_{f}$ is the quantity of frequency points).

However, the inverse problem suffers from ill-posed and ill-conditioning problems which make the optimization extremely sensitive to measurement noise. To stabilize the optimization, the regularization methods originated from Tikhonov impose the prior knowledge as a regularization term to Function (18), which functions as a spectral filter. The objective function becomes:

(19) $$\underset{v}{\min}f=\frac{1}{2}\|\Delta L\left(v\right)-\Delta L_{m}{\|}_{2}^{2}+\beta R\left(v\right)$$

where β is the relaxation factor and the term R(v) for $\ell_{2}$ regularization is $R\left(v\right)=\|v{\|}_{2}^{2}$.

The measurements in the objective function can be the impedance of the Rx coil [57] and magnetic field values from magnetic field sensors [58]. The corresponding calculated values are usually obtained from the analytical and numerical models. The number of independent measurement points affect the estimation results to some extent [59]. The numerical simulation is usually time-consuming, whereas the analytical models are only suitable for the homogenous isotropic materials. For the isotropic material in axisymmetric geometry, the Dodd and Deeds model and its simplification have been intensively investigated [26,60], while for the asymmetric model, the SOVP method can be applied [61]. To analyze the complex structures consisting of the heterogeneous electromagnetic property distribution [62] and anisotropic materials, for example, carbon fiber reinforced polymer (CFRP) [63], the FEM as well as hybrid FEM and boundary element method (BEM) can be adopted.

The combination of optimized variables influence the optimization performance. It is expected that the nonlinear objective function reaches its global minimum in the variable space, which only exists when the objective function is convex. This corresponds to the situation that $\nabla_{v}\nabla_{v}f≽0$. The second-order derivative, the Hessian matrix, is usually approximated by $\nabla_{v}\nabla_{v}f\approx H\left(v\right)={\left(\nabla_{v}f\right)}^{H}\nabla_{v}f$, and $S\left(v\right)=\nabla_{v}f$ is referred to as the sensitivity/Jacobian matrix. If the influences of various variables on measurements are correlated, for example, coupling effect of permeability and conductivity on inductance [64,65,66] and correlation between the derivatives in terms of thickness and electromagnetic parameters [61], the condition number of the Hessian matrix will be large, and the function convexity can hardly be satisfied. Furthermore, the variable correlation renders the optimization process unstable when the inversion of the Hessian matrix is required [67]. The appropriate variable combination can be determined according to local and global sensitivity analyses. The local sensitivity analysis focuses on the Jacobian matrix evaluated on specific points, for example, the singular value analysis, while the global analysis can reflect the correlations between variables, for example, the variance-based methods [68,69]. The ill-conditioning degree of the inverse problem can be represented by the singular value spectrum of sensitivity/Hessian matrix [70]. If there is no extremely small singular value, the optimization process would be stable and affected little by the measurement noise. The singular value feature of the Hessian matrix can be represented by $S_{f}:{\mathbb{C}}^{n_{v}\times n_{v}}\to \mathbb{R}$:

(20) $$S_{f}\left(H\left(v_{r}\right)\right)=-20\log_{10}\left(\left|\lambda_{\min}|/\lambda_{\max}\right|\right)$$

where $\lambda_{\min}$ and $\lambda_{\max}$ are the minimum and maximum singular values of $H\left(v_{r}\right)$, respectively.

In the optimization process, the variables can be updated by various strategies according to the gradient of the nonlinear objective function. The representative methods and their characteristics are shown in Table 3. The variables are updated in an iterative manner:

(21) $$v_{k+1}=v_{k}+\Delta v_{k}$$

For a small number of optimized variables, the heuristic methods which employ various solution searching strategies, including classic genetic algorithm (GA) and particle swarm optimization (PSO), are usually implemented in conjunction with other methods. These methods decrease the influence of starting point selection meanwhile improve the algorithm’s convergence and maintaining its correctness as long as the heuristic is admissible [71,72].

In previous studies, the electromagnetic properties and thickness of metallic plates have been estimated by the modified Newton-Raphson method [73]. The aforementioned algorithms have been evaluated in the reconstruction of conductivity profile in the graphite blocks [56,74]. In addition, the reconstruction of defects in the anisotropic materials employing the GA method has been proposed in the study [63]. The shape reconstruction of conductive clogging deposits in the steam generator is realized in research [62], using the gradient descent method employing the shape derivatives.

## 3. Measurement System

### 3.1. Impedance Measurement System

#### 3.1.1. Overview of EC Testing Instruments

EC testing techniques are widely applied in various industrial applications [27]. In practice, the system configuration, e.g., hardware and software implementation, differs from instrument to instrument and highly depends on each particular application. In general, the system contains an excitation source, which can be pulsed excitation signal [75], single frequency signal [28,76,77] and multi-frequency signal [78,79,80,81,82], etc. The hardware, data acquisition system (DAQ), normally includes analogue and digital conditioning electronics, which implement signal generation, signal amplification, demodulation and filtering. Typically, a control unit is in charge of the operation in the hardware, which can be a digital signal processor (DSP), a field programmable gate array (FPGA) or a microcontroller, etc. For software, normally it varies from the programming language for the user interface such as C or C++ [83,84] and LabVIEW graphical programming language [81,85,86]. Due to different requirements of measuring speed, the electromagnetic (EM) instruments can also be divided into on-line or off-line instruments [87]. In addition, there are more variations when the system is interfaced to sensors of different configurations, such as the electromagnetic tomography (EMT) sensor array, which requires multiplexing operation of the instrument [88,89].

**Figure 8:**
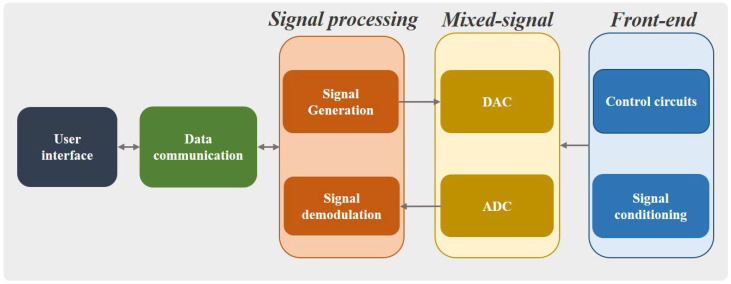
Generic block diagram of EM instruments.

Although much work has been carried out for EM system development, there is still a further expectation in improving hardware and software system design. With the development of technology and higher demand in the industry, the hardware and the software have kept advancing. The development of analogue EM systems has a long history, which relies on constructing physical circuits by using electronic components as in [89,90]. Compared with analogue systems, digital systems are of increasing interest as they are more reliable, robust and less complex in hardware configuration [83]. The digital systems commonly apply a DSP or an FPGA as cornerstone, which can process the detected signals digitally and quickly. Generally, a digital instrument consists of a processing unit, a control logic, front-ends and possibly a power management part. A generical block diagram of digital system is shown in Figure 8.

#### 3.1.2. Customized EC Testing Instrument

The EC testing instruments have been developed for various measurement applications. The recent studies and system characteristics are summarized in Table 4.

**Figure 9:**
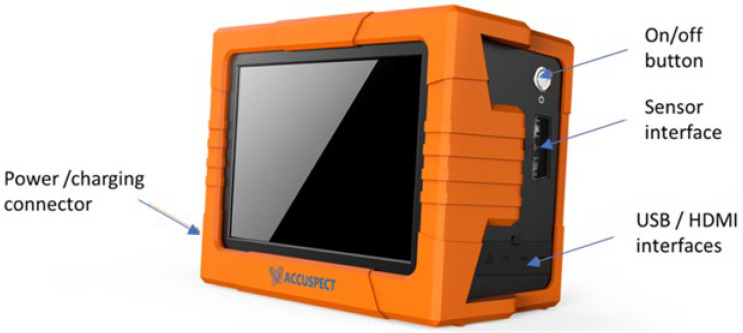
Self-designed and constructed EC testing instrument.

The EC testing instrument developed in the EM sensing group led by W. Yin at the University of Manchester is shown in Figure 9. The cornerstone of the instrument is an FPFA SoC, which integrates an ARM dual Cortex-9-based processor and a Xilinx 7-series FPGA. The FPGA module is responsible for excitation signal generation, implementation of in-phase and quadrature phase (IQ) demodulation, multiplexing control, and data transfer. Generally, the FPGA is preferable for implementing a high-speed logic, arithmetic, and dataflow subsystem. Therefore, it is suitable for real-time online detection and imaging of defects, which requires high data speed. The ARM processor is in charge of data transfer and communication. With ethernet communication, the instrument has a fast and robust transmission, and it can provide a data rate up to 10 k samples/s. Under this configuration, the interference between the FPGA and the processor is minimized.

**Figure 10:**
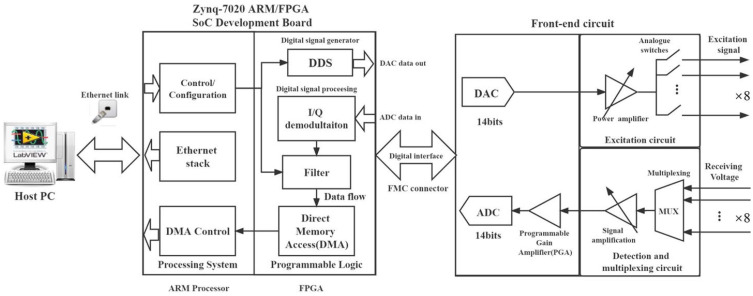
System architecture of the customized EC testing instrument.

A block diagram of the system architecture is shown in Figure 10. In addition to the SoC, the instrument also consists of front-end circuits including excitation circuits, detection circuits, and mixed-signal circuits. Depending on the probe structure, it may also involve multiplexing when using multi-channel probes. The excitation circuit generates a sinusoidal signal to energize the Tx coils, and the detection circuit receives the induced voltage on the Rx elements. The excitation frequency ranges from 1 kHz to 1 MHz, which is applicable for most EC testing applications. A typical loop-back signal-to-noise ratio (SNR) can reach up to 100 dB. However, the real SNR including sensor elements depends on the measurement configuration.

A built-in user interface running on the host PC was also developed. The user interface provides a convenient way to display and log data. It also allows the user to send configurations such as operating frequency, filter width, and coil pair assignments during the experiments. After measurements, the data is saved in a database format (.db), which can be directly fed into further signal processing algorithms.

The instrument integrates all elements in a case with a size of 288 mm × 208 mm × 135 mm, which makes it portable for in-situ tests. There are also USB and HDMI interfaces that can connect to external devices. Notably, the instrument can operate with or without the power connection as it includes an internal battery with automatic power management and can last for more than 8 h during typical use once fully charged.

#### 3.1.3. Commercial Impedance Analyzers

**Figure 11:**
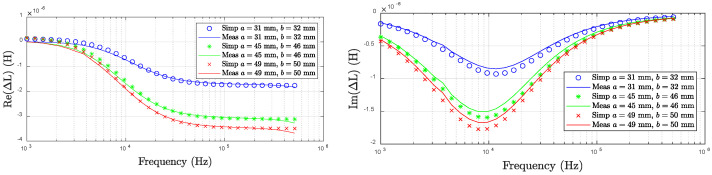
Frequency responses of eddy current probes.

Impedance is an important parameter characterizing the frequency response of EC probes. In the analytical solution of Dodd and Deeds, the inductance of Rx coil is calculated as the result in the presence of test pieces [13,14]. Generally, impedance is defined as the total opposition a device or circuit offers to the flow of an alternating current at a given frequency and is represented as a complex quantity [98]. The impedance vector consists of a real part R and an imaginary part X. The real part represents the resistance while the imaginary part denotes reactance which can be either inductive or capacitive. To acquire the impedance, both the real part and the imaginary part need to be measured [98]. The representative frequency responses of the imaginary and real parts of EC probes are shown in Figure 11.

As compared with using off-the-self commercial impedance analyzers, the development of customized instruments can be complicated and time-consuming. Therefore, commercial impedance analyzers play essential roles in laboratory-based experiments of EC testing as they may have wider operating ranges and better performances in terms of accuracy and precision than customized instruments.

**Figure 12:**
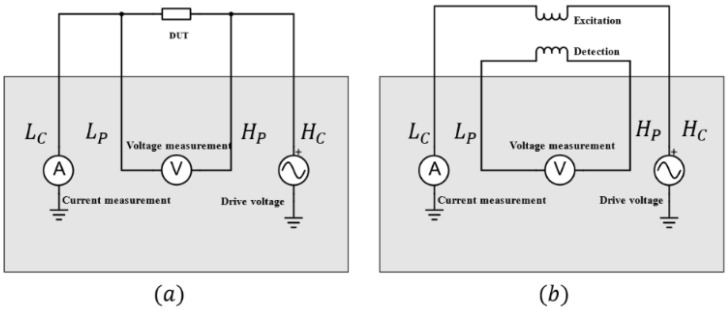
Terminal arrangement of commercial impedance analyzers for: (**a**) impedance measurement of DUT; (**b**) mutual inductance measurement between the excitation and detection coils.

Typically, commercial impedance analyzers implement 4-port measurement, where both voltage and current are measured. The terminal arrangements are shown in Figure 12a,b when measuring a single element and mutual inductance between coils, respectively. Table 5 illustrates the characteristics of two commercial impedance analyzers that have been intensively used in previous studies [99,100].

#### 3.1.4. Calibration

Generally, electronic components such as op-amps introduce phase shifts at different frequencies in acquisition channels. To reduce these phase shifts, calibration is normally required for the measuring system. Typically, it can be achieved by taking a set of measurements using a small permeable or conductive object in air space. As the expected signal in these cases has only a real (or imaginary) component, the phase shift can be manually adjusted for each frequency to minimize the imaginary (or real) component. Then, the obtained phase for each channel can be stored and subtracted automatically from all posterior measurements using other objects [101]. A conductive brass sphere was used in [101], which was supposed to only provide a real component. In [86,102], a ferrite object was used for calibration, which was magnetically permeable but not electrically conductive. Therefore, the measurements should only change along the imaginary part in the complex plane. In this way, the errors and phase shifts introduced in the acquisition channels can be effectively attenuated.

**Figure 13:**
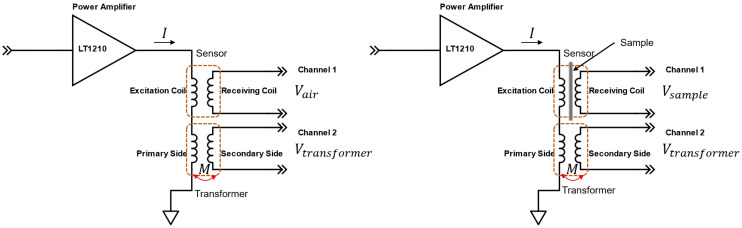
Calibration with a current sensing transformer.

An alternative calibration method using a current sensing transformer was proposed in [103], where both induced voltage and excitation current were measured. As compared with current sensing resistors, it provides a better dynamic range when employing coil probes. Furthermore, the transformer has quite a flat magnitude response under different frequencies and introduces little phase shift between the primary side and the secondary side. The diagram of calibrations is shown in Figure 13, with a current sensing transformer. The relative inductance in the presence of a sample can be calculated by using Equations (22) and (23):

(22) $$V_{\mathrm{transformer}}=j\omega MI$$

(23) $$\Delta L=\frac{V_{\mathrm{sample}}-V_{\mathrm{air}}}{j\omega I}=\left(V_{\mathrm{sample}}-V_{\mathrm{air}}\right)\times \frac{M}{V_{\mathrm{transformer}}}$$

where $V_{\mathrm{sample}}$ is the induced voltage in the Rx coil in the presence of a sample, $V_{\mathrm{air}}$ is the induced voltage in the Rx coil in free space, $V_{\mathrm{transformer}}$ is the induced voltage at the secondary side of the transformer, M is the mutual inductance between the primary side and secondary side of the transformer, I is the excitation current, and ω is the angular frequency of the excitation signal.

### 3.2. Probe Design

**Figure 14:**
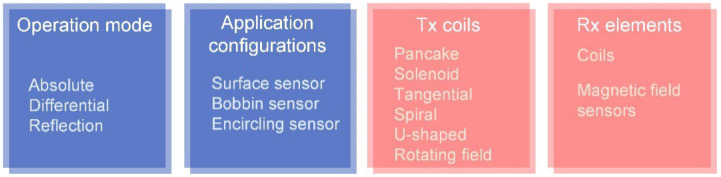
Categories of the eddy-current probes.

The EC probes can be categorized according to the operation mode and configuration of applications [104,105], as shown in Figure 14. The EC probes and related signal processing techniques have been summarized in previous studies [29,30,106,107,108]. The characteristics of the conventional probes are briefly analyzed below.

For the operation modes, the absolute mode usually measures the absolute impedance of Rx coil, which reflects the thickness, conductivity, permeability, and defect information; the differential mode measures the defect on the test pieces using the differential signal of coils that is insensitive to the slowly varying properties and environmental influences; the reflection mode conventionally referred to as driver/pickup mode adopts the Tx and Rx coils which are optimized separately for various purposes.

In the reflection mode, the Tx and Rx coils are considered separately. There exist various structures of Tx coils, for example, pancake, tangential, planar, and U-shaped/U-cored coils. The basic structures of several representative Tx coils and sensitive regions on measured plates are illustrated in Table 6.

To measure thick plates and pipes, the EC penetration depth is limited by:

(24) $$\delta =\frac{1}{\sqrt{\pi f\mu \sigma }}$$

The defined penetration depth corresponds to the planar wave excitation and is usually higher than that of the practical Tx coils in finite dimensions. The normalized standard penetration depth can represent the influence of variables in Equation (24) [109], i.e., excitation frequency, conductivity, and permeability. The factors that affect the penetration depth including the coil dimensions have been summarized in the study [110]. It has been suggested that the tangential coil is more suitable for the thick plate measurement, as the induced EC of the widely adopted pancake coils suffers from the diffusion and axial cancellation effect, resulting in more obvious longitudinal EC decay [109,111,112].

The U-shaped coil usually contains a ferrite core that constitute the magnetic circuit and concentrates the magnetic field beneath the two ends, which increases the signal amplitude caused by the defects [113,114,115]. The planar coil fabricated by flexible printed circuit (FPC) and printing electronic technology (PET) elevates the detection capability of micro-surface defects [116,117]. Furthermore, by employing a suitable track width of the planar spiral coils, the coil impedance consists of both the capacitance and inductance, reflecting various electromagnetic properties simultaneously [118]. To measure the defects in an arbitrary direction, it is efficient to employ a rotating magnetic field probe composed of several Tx coils with different excitation signal phase and positions [104,119,120,121,122]. The most significant variation of EC field due to the crack occurs when it is perpendicular to the defect direction.

The Rx elements include coils and magnetic field sensors which measure the coil impedance and magnetic field, respectively. In comparison, the coils are characterized by their linear response, no saturation, small hysteresis, and high flexibility, while the magnetic field sensors, for example, Hall sensor, anisotropic magneto resistor (AMR), giant magneto resistor (GMR), tunneling magneto resistance (TMR), and superconducting quantum interference device (SQUID), are in small dimensions and capable of low-frequency measurement in the sensitive direction. Nevertheless, the magnetic field sensors are limited in the dynamic range, to some extent, and provide the nonlinear response signal. The detailed characteristics and comparison of the magnetic field sensors are summarized in the literature [123,124,125]. Increasing the number of Rx elements accounts for an increase in the independent measurement signals, thereby, enriching the prior information to estimate the physical properties of objects. The plate thickness and permeability, for instance, can be derived using the single frequency inductance from multiple Rx coils with the simplified analytical model [99,126,127]. Furthermore, to better the accuracy solving the inverse problem, the optimum design of coil array has been discussed for electromagnetic tomography (EMT) [128,129], while few studies have considered this issue in plate measurement applications.

Probe shielding reduces the interaction of the exciting magnetic field with the nonrelevant region in proximity of the probe, which is usually applied to reduce edge effects. In addition, probe loading employs the ferrite cores which are ferromagnetic concentrating the magnetic field for the sensing area. These strategies are significant, especially for remote field EC testing [130] and for reducing the size of the device.

The EC probes can integrate with the sensors of other sensing modalities, for example, capacitance electrodes. An integrated sensor can obtain complementary information and is sensitive to more electromagnetic properties of the test pieces. A dual-modality inductive and capacitive sensor facilitates plate thickness measurements using the single frequency inductance [131], as the lift-off can be directly derived from the capacitance between the surfaces of probe and plates.

## 4. Planar Structure Measurements and Applications

### 4.1. Reducing the Lift-Off Effects

The EC testing of planar structure suffers from the lift-off effect, which attenuates the magnitude and changes the phase of measured signals, causing errors in property estimation. To address this issue, researchers have investigated a range of methods including compensation strategies, algorithms, and lift-off invariance phenomenon.

#### 4.1.1. Lift-Off Compensation Using Multiple Measurements

Analytical models are frequently adopted to estimate plate properties, due to explicit expressions and fast calculations. Although asymmetric problems exist in applications, a variety of cases of planar structure measurement can be approximated using the axial-symmetric model employing the Dodd and Deeds model with adequate accuracy.

**Figure 15:**
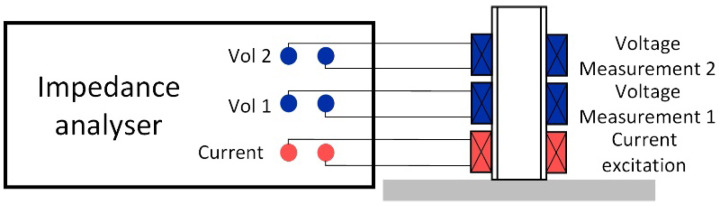
Measurement configuration for lift-off compensation employing triple-coil probe.

In the Dodd and Deeds model, the plate term of the coil inductance is a function of excitation frequency, spatial frequency, and plate properties. In the approximate model expressed by Equation (5), the characteristic spatial frequency $\alpha_{0}$ of coil probes is affected by the lift-off. The compensation methods estimate the lift-off and calculate the compensated characteristic spatial frequency $\alpha_{0r}$, then, a specific plate property can be estimated from the phase of inductance. As the unknown parameters are lift-off and plate property, the measurements of various Rx coils (or different positions of single Rx coil) are implemented to solve the linear equations of the simplified analytical model. The representative measurement configuration is shown in Figure 15.

For the nonmagnetic plates, according to the model in Equation (5), the phase of inductance is determined by the plate term $\phi \left(\alpha_{0}\right)$. In the low excitation frequency, the approximation that $\alpha_{1}c\to 0$, $e^{2\alpha_{1}c}\sim 1+2\alpha_{1}c$ can be satisfied. Consequently, $\phi \left(\alpha_{0}\right)$ can be simplified to [114]:

(25) $$\begin{array}{c} \phi \left(\alpha_{0}\right)\approx \frac{j\omega \sigma \mu_{0}c}{j\omega \sigma \mu_{0}c+2\alpha_{0}{}^{2}c+2\alpha_{0}+2\alpha_{0}\alpha_{1}c}\\ =\frac{j\frac{\omega }{\omega_{0}}}{1+\frac{2\alpha_{0}\alpha_{1}c}{2\alpha_{0}{}^{2}c+2\alpha_{0}}+j\frac{\omega }{\omega_{0}}} \end{array}$$

where $\omega_{0}=2\alpha_{0}/\left(\sigma \mu_{0}c\right)$.

When the plate thickness c is a small value, the plate term can be simplified by the first-order system [100], of which the peak frequency is $\omega_{0}$, i.e.:

(26) $$\phi \left(\alpha_{0}\right)\approx \frac{j\omega /\omega_{0}}{1+j\omega /\omega_{0}}$$

The simplified plate term indicates that the plate thickness is inversely proportional to the peak frequency of imaginary part of inductance [114]. Providing the characteristic spatial frequency and phase of inductance, the plate thickness can be estimated using Equation (26).

Alternatively, for the magnetic plates employing the high excitation frequency ($\alpha_{1}c\to \infty$), the plate term in Equation (2) can be approximated by [126,132,133]:

(27) $$\phi \left(\alpha_{0}\right)\approx -\frac{\alpha_{1}-\mu_{1}\alpha_{0}}{\alpha_{1}+\mu_{1}\alpha_{0}}$$

Applying the approximation $\alpha_{1}=\sqrt{\alpha^{2}+j\omega \mu_{0}\mu_{1}\sigma }\approx \sqrt{j\omega \mu_{0}\mu_{1}\sigma }=\left(1+j\right)\;\alpha ’,\;\alpha ’=\sqrt{2\omega \mu_{0}\mu_{1}\sigma }/2$, Equation (27) can be approximated by:

(28) $$\phi \left(\alpha_{0}\right)\approx -\frac{2\alpha {’}^{2}-\mu_{1}{}^{2}\alpha_{0}{}^{2}+j2\mu_{1}\alpha_{0}\alpha ’}{{\left(\alpha ’+\mu_{1}\alpha_{0}\right)}^{2}+\alpha {’}^{2}}$$

In this way, the real part of the plate term relating to the real part of inductance can be simplified as:

(29) Re[ϕ(α0)]≈−2α’2−μ12α02(α’+μ1α0)2+α’2=2μ12α02+2μ1α0α’2α’2+μ12α02+2μ1α0α’−1

Since $\alpha ’\gg \alpha_{0}$ for the high excitation frequency, the real part can be approximated by [126]:

(30) Re[ϕ(α0)]≈μ1α0α’−1=2μ1ωμ0σα0−1

The relative permeability can be estimated if $\alpha_{0}$ is obtained. For the unknown lift-off, the compensated characteristic spatial frequency $\alpha_{0r}$, relating to the maximum value in the sensor term series, is calculated by [17]:

(31) $$\alpha_{0r}=\alpha_{0}-\frac{4\alpha_{0}^{2}l_{0}}{\pi^{2}}$$

For the plates in a large radius, according to Equation (8), the inductance magnitude can be calculated by:

(32) $$\begin{array}{c} L_{0}\approx A_{m}\left(\alpha_{0}\right)\int_{0}^{2\alpha_{0}}e^{-2\alpha l_{0}}\sin^{2}\left(\frac{\alpha \pi }{2\alpha_{0}}\right)d\alpha \\ =A_{m}\left(\alpha_{0}\right)\frac{\pi^{2}\alpha_{0}\left(1-e^{-2\alpha_{0}l_{0}}\right)}{2\alpha_{0}l_{0}\left[{\left(\alpha_{0}l_{0}\right)}^{2}+\pi^{2}\right]} \end{array}$$

where $l_{0}$ indicates the lift-off of Rx coil and the upper limit of integral relates to the range of the first peak of the sensor term, as shown in Figure 2.

In Equation (32), the linear relationship between the lift-off $l_{0}$ and magnitude of inductance $L_{0}$ can be obtained through simplification. The relationship can be applied to estimate $l_{0}$ from the inductance measured by Rx coils [17]. Combining Equation (25) for low excitation frequency, or Equation (30) for relatively high frequency and inductance magnitude, the thickness and lift-off can be estimated for nonmagnetic plates [99,100,114,126], while the permeability and lift-off can be calculated for the magnetic plates [24,132]. The experimental evaluation of aforementioned methods are shown in Table 7.

In addition, it is observed that a series of quadratic-like curves related to the plate thickness can approximately describe the relationships between the phase and logarithmic amplitude performing the lift-off scan with the inductor–capacitor (LC) resonant probe. The plate thickness can be estimated employing the curve fitting method [134].

#### 4.1.2. Lift-Off Compensating Algorithms

A variety of algorithms have been proposed to address the influence due to probe lift-off on the electromagnetic property estimation of planar structures. W. Yin et al. proposed the characteristic frequency which relates to the thickness and conductivity of the planar plate [33]. Furthermore, it was observed that the characteristic frequency decreased with an increase in the lift-off. By utilizing the triple-coil probe with fixed coil separations, the change of characteristic frequency under the range of lift-offs tested remained a constant value [100]. Avoiding the requirement for precise magnetic balance, the compensation algorithm was proposed to tackle the lift-off variation by using the compensated peak frequency feature. Accordingly, the accuracy was increased while the mechanical configuration was simplified [17]. Furthermore, M. Lu et al. employed the zero-crossing frequency of the real part of inductance to inspect the properties of both nonmagnetic and magnetic materials [135]. The magnetic permeability prediction approach was given based on the phase compensating algorithm to reduce the inaccuracy of impedance phase for ferrous steels caused by the probe lift-offs. The error in permeability prediction was less than 2% within the evaluated range [132]. In addition, H. Wang et al. proposed that the slope of the lift-off curve (LOC) in the RL impedance plane could characterize the target thickness which was independent of lift-off variation [136,137]. 

For heterogenous plates, the material property profiles have been reconstructed from frequency sweeping measurements [24,26,58,138]. By adopting the iterative optimization algorithms or simply fitting the EM measurements calculated by analytical models, the profiles for test pieces have been reconstructed. For magnetic materials, the induced eddy current concentrates near the surface of plates, due to the thin skin effect. Based on this effect, the simplified material-independent algorithm was derived to estimate the probe lift-off and plate permeability [24]. In [99], the proposed algorithm employed the inductance phase of two Rx coils in a single low excitation frequency to infer the probe lift-off, which was applied to compensate the estimated thickness of nonmagnetic plates. These compensation algorithms increased the lift-off tolerance to about 10 mm, while maintaining the high accuracy of plate property estimation.

#### 4.1.3. Lift-Off Invariance Phenomenon

**Figure 16:**
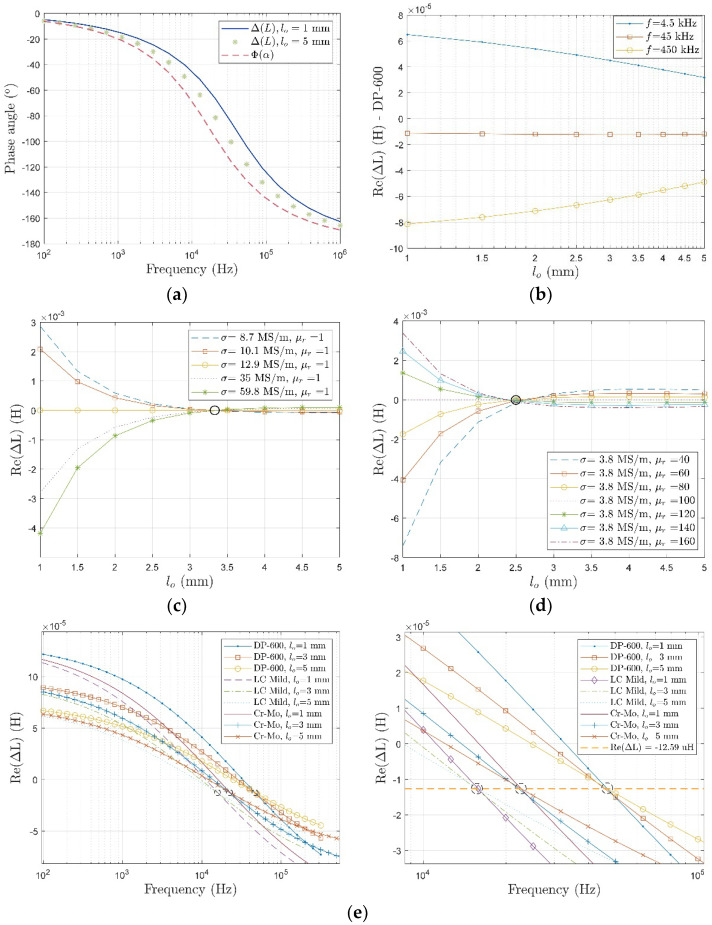
Lift-off invariance features: (**a**) Phase invariance phenomenon; (**b**) lift-off invariance inductance; (**c**) conductivity invariance phenomenon; (**d**) permeability invariance phenomenon; (**e**) lift-off invariance frequency.

Measurements based on the electromagnetic induction method are sensitive to probe lift-off. In an investigation attenuating the lift-off effect, the lift-off invariance phenomenon was observed and applied to measure the conductive plates. As shown in Figure 16, it was found that at a certain range of lift-off, there existed a certain lift-off that made the influence of the conductivity/permeability on the inductance unchangeable, which was termed the conductivity invariance phenomenon (CIP) [139] and permeability invariance phenomenon (PIP) [140]. This phenomenon can decouple the correlation between electrical conductivity and magnetic permeability at an optimal lift-off. In [141] and [142], the authors found that the phase curves of measurements for different lift-offs maintained a stable value. H. Wang et al. observed that, for a large distance of lift-off, the approximate linear relationship held between the logarithms of the phase signal and the plate conductivity [136]. C.S. Angani et al. indicated that the magnetic field measurements of various lift-offs intersected at a certain point in the frequency domain when performing the transient EC oscillation method [143].

With the aid of LII and LIF, optimization methods have been applied to calculate plate properties.

Under high excitation frequency, it has been proposed that there exists a linear relationship between the ratio of inductance change and the probe lift-off, namely the dual-frequency linearity of lift-off (DFL) feature [133]. This can be formulated as:

(33) $$\;l_{0}=\frac{5.56\left(4\alpha_{0}{}^{2}{\left(g+h_{c}\right)}^{2}+\pi^{2}\right)\left(g+h_{c}\right)\Delta L_{2}}{\pi^{2}\left(1-e^{-4\alpha_{0}\left(g+h_{c}\right)}\right)\Delta L_{1}}-\frac{1.39}{\alpha_{0}}$$

where g and $h_{c}$ are gap between coils and the height of coil, respectively.

It has been noticed that there was a lift-off invariance inductance (LII) for different samples under various excitation frequencies, termed the lift-off invariance frequency (LIF) [144]. The estimation of plate properties employing the inductance measurement phenomenon is summarized in Table 8.

### 4.2. Defect Scanning

**Figure 17:**
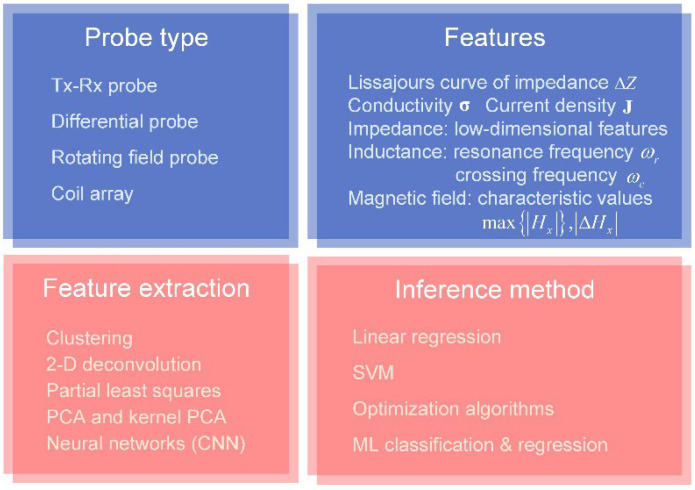
Key factors estimating the defect information.

The defect evaluation is one of the major applications of the EC system in metallic plate measurement. The measurement features of signals in the time and frequency domain are extracted to infer the defect information. The recent research highlights are shown in Figure 17. Representative probes, measurement features, and inference methods are illustrated below.

**Figure 18:**
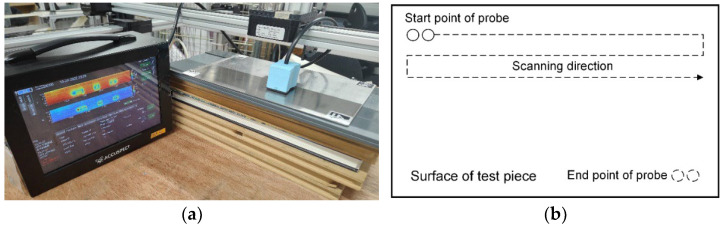
(**a**) Experimental setup of EC system; (**b**) diagram of defect scanning.

**Figure 19:**
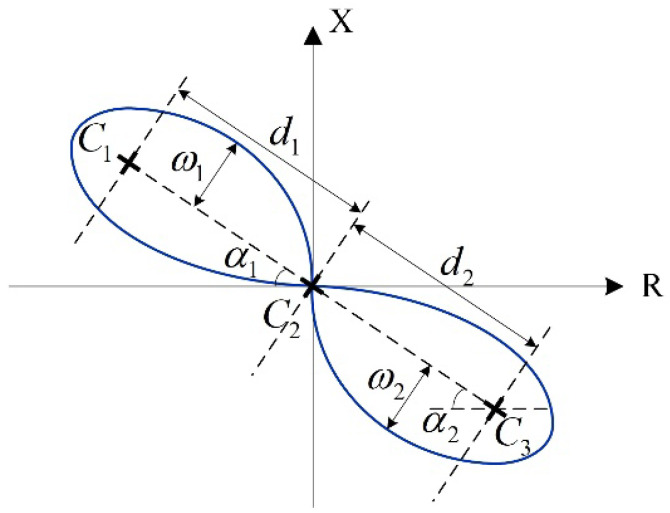
Geometric features of the Lissajous curve in the impedance plane. $C_{1}$, $C_{2}$, and $C_{3}$ are clustered centers of a variety of impedance curves.

The probes suitable for the surface defect measurement include the Tx and Rx coils as well as magnetic field sensors. The measured time series and frequency spectrum when scanning the probe above the test pieces, as shown in Figure 18, reflects the properties of defects. The experiments indicate that the probe is sensitive to the defect especially when the induced eddy current is perpendicular to the defect orientation [145]. Accordingly, the rotating field coils, constructed by the planar and ferrite-core coils, have been designed to generate the magnetic field for the measurement of crack directions [146,147]. The signals of differential probe are featured as the so-called Lissajous curve, as shown in Figure 19, of which the geometric features reflect the depth of defects [148]. In addition, the probe lift-off affects the signals of Rx elements, and the influence is expected to be a fixed value during the scanning process [147]. In addition, the variation of scanning signals due to lift-off can be applied to estimate the surface corrosion of plates [149].

The defect information can be obtained from electromagnetic measurements and corresponding statistical features. The electromagnetic measurements are measured from coils and magnetic field sensors. The statistical features are obtained from the electromagnetic measurements through the linear and nonlinear feature extraction methods of machine learning and pattern recognition, which is intended to be informative and nonredundant, facilitating the subsequent estimation of plate properties and defect information.

**Figure 20:**
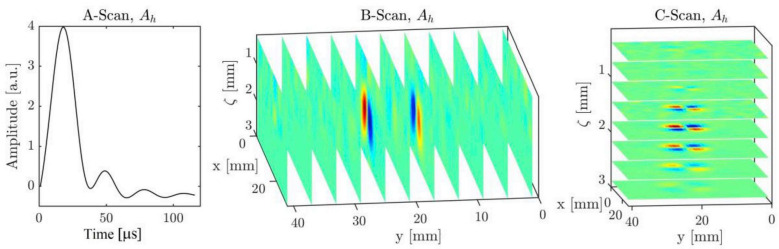
Various scanning modes where A-Scan is the pulse response of a single point; x, y, and ζ indicate the width, length, and depth of a rectangular plate, respectively [154].

**Figure 21:**
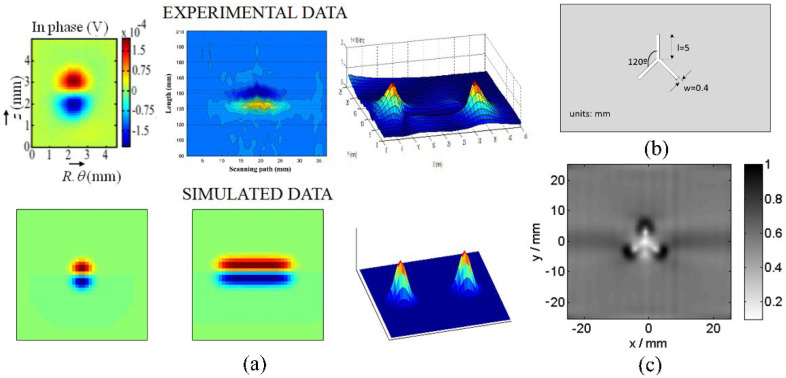
(**a**) Comparison between the measured magnetic field signal (experimental data) and HG_0.1_ pattern (simulated data) [155]; (**b**) shape of defect; (**c**) normalized sum of Eddy current density distributions [147].

The measurements of coil at the characteristic frequencies, for example, peak and zero-crossing frequencies, contain the information of plate properties and probe lift-off, while the pattern of measured magnetic field parameters relates to the geometry of defects. It has been observed that the crossing frequency of a specific real part of the coil inductance corresponds to the permeability, which relates to the hardness of magnetic materials [150]. The lift-off effect of probe employing magnetic field sensors can be compensated for in the Fourier domain [151]. Furthermore, the conductivity distributions reconstructed in the ROI by inverse methods can represent the arbitrary defect location and shape [88,152]. Through the various scanning modes shown in Figure 20, it has been proposed that among the features of |H(f,x,y)|, Arg{H(f,x,y)}, |Γ(t,x,y)|, and Arg{Γ(t,x,y)} (Γ(t,x,y)=H(t,x,y)+j{H(t,x,y)}, {·} represents the Hilbert transform) and Arg{Γ(t,x,y)} can characterize the defects in relatively deeper locations of test pieces [153]. The distribution of the measured magnetic field approximates to the Hermite-Gaussian (HG) modes which are solutions of analytical function [154], i.e., paraxial wave equations in Cartesian coordinates [155]. Furthermore, the Biot–Savart law implies that the variation of EC distribution reflecting the defect contour, as shown in Figure 21, can be approximated by the 2D deconvolution of magnetic field change and dipole current map [147,154], since:

$$\left[B_{x}]=[\mathrm{conv}\left(\left[b_{x}],[J_{d}\right]\right)\right]$$

where $\left[B_{x}\right]$ is a matrix that maps the magnetic field perturbation, $\left[J_{d}\right]$ is the matrix that maps the EC perturbation induced in the metallic surface, $\left[b_{x}\right]$ is a transfer matrix that represents the field produced by a unit dipole current, and conv(·,·) indicates the 2D convolution operation.

The statistical features of measurements in lower dimensional space can be extracted employing the linear methods including principal component analysis (PCA), partial least squares (PLS) [156], clustering methods [148], and nonlinear methods, for example, kernel PCA [157] and neural networks of machine learning (ML) [158,159]. The no-linear mapping from the statistical features to defect dimensions is usually modeled implicitly by ML. In ML models, it is expected that the measurement features of training and test samples are uniformly distributed in a similar region of the lower dimensional space. This implies that the independent identical distribution hypothesis of samples can be satisfied, so that the high prediction accuracy and generalization can be achieved. The adaptive sampling scheme, for esample, output surface filling can be adopted to realize this condition.

Specifically, the discrete defect depth and lift-off can be estimated by classification methods, for example, support vector machine (SVM) and other ML models, implementing the classification loss functions, while the continuous dimensions and shape of defects can be obtained by the regression models [156].

Although the emerging deep learning (DL) models have demonstrated advantages regarding defect reconstruction and lift-off tolerance [160], the robustness of DL models is still one of the primary challenges. For instance, when measuring anisotropic ferromagnetic plates and unknown materials, the prediction errors tend to increase, due to large discrepancies between the available training samples prepared beforehand and test samples [161]. In the on-line monitoring process where the estimation speed and safety are in high requirement, it is difficult to deploy the large-scale DL models. The DL method for defect scanning still requires investigation of the state-of-the-art models, dataset construction, and deployment in various conditions, to test its performance and reliability.

## 5. Conclusions

In this review, we summarize the recent developments of EC testing for planar structures. The key issues in computational models, measurement systems, and parameter estimation are summarized. In the theoretical analysis, the forward and inverse problems are described, elaborating the basic principles of the analytical model and FEM for the forward problem as well as simplification and optimization algorithms for the inverse problem. The system development consists of a customized and commercial grade impedance measurement system including the calibration method, and probe design which is task specific. In applications of plate property measurement, the lift-off compensation methods are addressed. Furthermore, in defect scanning, the joint application of probes, measurement feature extraction, and defect parameter inference methods are investigated.

Computational models are being developed for complex geometry and real-world applications, while, at the same time, simplified methods increase the speed of computation for real-time measurements. The sensitivity and spatial resolution of measurements can be improved by system improvements as well as customized and application-specific probe optimization. Lift-off tolerance/elimination methods are still topics worth investigation. In summary, the combination of cutting-edge models and high-performance measurement systems is still required and needs to be explored through continuous efforts for many existing and potential industrial applications.

## Figures and Tables

**Table 1:** The computation and accelerated times under different mesh elements [22].

Element Number	Calculation Time of the Method without Acceleration (s)	Calculation Time of the Proposed Method (s)	Accelerated Rate (Times)	Calculation Deviation (%)
10 k	7.69	2.03	3.79	2.56
51 k	57.74	3.56	16.22	3.22
139 k	306.79	8.96	34.24	3.56

**Table 2:** Approximate linear relationship between the endpoint of inductance trajectory (marked by circles) and tilt angles [53].

#	Copper	Aluminium
Inductance trajectory	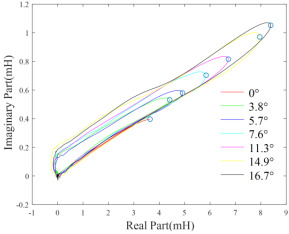	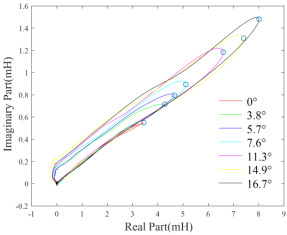

**Table 3:** Characteristics of nonlinear optimization algorithms.

Algorithm	Updating Direction, $\Delta v_{k}=d_{k}\left(\Delta L-\Delta L_{m}\right)$	Convergence Rate	Stability
Gradient descent	$d_{k}=-S_{k}^{H}$	Slow	High
Conjugate gradient	$d_{k}=-S_{k}^{H}$+$\beta_{k}d_{k}$, $\beta_{k}=\max\left\{0,S_{k}^{H}\frac{\left(S_{k}-S_{k-1}\right)}{S_{k}{}_{2}^{2}}\right\}$	Fast	Medium
Newton method	$d_{k}=-{\left(S_{k}^{H}S_{k}\right)}^{-1}S_{k}^{H}$	Fast	Low
LM	$d_{k}=-{\left[S_{k}^{H}S_{k}+\beta \mathrm{diag}\left(S_{k}^{H}S_{k}\right)\right]}^{-1}S_{k}^{H}$	Medium	Medium
PSO	$\Delta v_{k}=\tau \Delta v_{k-1}+c_{1}rand_{1,k}\left(v_{\mathrm{pBest},k}-v_{k}\right)$ $+c_{2}rand_{2,k}\left(v_{\mathrm{gBest},k}-v_{k}\right)$τ is an inertial constant, $c_{1}$ and $c_{2}$ are step length, $rand_{1}$ and $rand_{2}$ are random variables, and $v_{\mathrm{pBest}}$ as well as $v_{\mathrm{gBest}}$ indicate the estimated local and global optimum.)	Medium	High

**Table 4:** Characteristics of customized EC testing instruments in recent studies.

Researcher	Controller	Applied Excitation Frequency	SNR	Software	Main Application
M. Kekelj et al. [91]	Cyclone V SoC	100~400 kHz	Up to 56 dB	MATLAB	Pipe defects
M. Hamel et al. [92]	NI DAQ	50~150 kHz	-	LabVIEW	Plate with crack
N. Zhang et al. [93]	NI DAQ	0.1~1 kHz	-	MATLAB	Cylindrical samples
A. K. Soni et al. [94]	NI DAQ with lock-in amplifier	0.5~80 kHz	38 dB	LabVIEW	Plate with crack
D. E. Aguiam et al. [95]	ADSP	Up to 10 MHz	-	LabVIEW	Block with crack
G. Zhang et al. [96]	NI DAQ with lock-in amplifier	50 kHz	-	LabVIEW	Plate with crack
G. Dingley et al. [97]	AVR MCU	0.1~100 kHz	Up to 90 dB	-	EMT

**Table 5:** Comparison of two commercial impedance analyzers.

-	Basic Accuracy	Frequency Range	Impedance Range	Data Speed	No. of Channels
Zurich MFIA 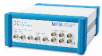	0.05%	1 mHz to 500 kHz/5 MHz	1 mΩ to 1 TΩ	20 msec/point for f > 10 kHz	1 Current, 1 Voltage
Solartron 1260A impedance analyzer 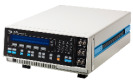	0.1%, 0.1°	10 µHz to 32 MHz	100 mΩ to 100 TΩ	No specified	1 Current, 2 Voltage

**Table 6:** Transmitting coils and corresponding magnetic fields on the surface and longitudinal section of measured plates.

Coil Structure	Magnetic Field Distribution, Normalized |H|	Coil Structure	Magnetic Field Distribution, Normalized |H|
Pancake coil 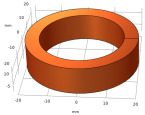	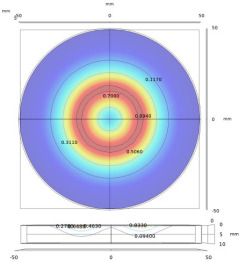	Spiral coil 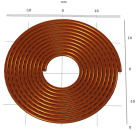	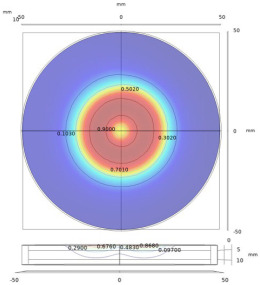
Solenoid coil 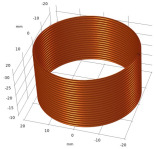	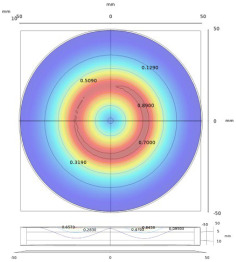	U-shaped (ferrite core) coil 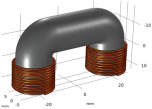	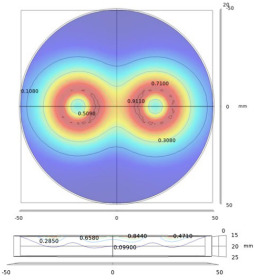
Tangential coil (rectangular) 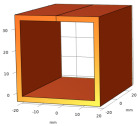	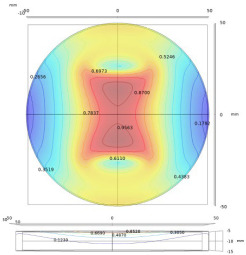	Rotating field coils 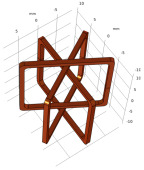	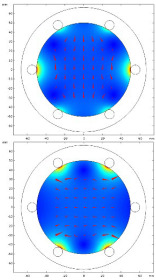 (Varying with time)

The color from blue to red indicates intensity from low to high.

**Table 7:** Experimental evaluation results of representative probes for lift-off compensation.

Probe Type	Plate Materials	Applied Frequency for Estimation	Lift-Off (mm)	Estimated Variables and Range	Relative Error of Estimation
Triple-coil [100]	Al	Peak frequency	Up to 6	Thickness (22~100 um)	<5%
Triple-coil [132]	DP 800, DP 1000	Zero-crossing frequency	Up to 4	$\mu_{r}$	<2%
Triple-coil [133]	DP 600, Cr-Mo	Dual frequency	Up to 20	$\mu_{r}$	<4.5%
Triple-coil [126]	Al, Cu	Single frequency (16 kHz)	Up to 5	Thickness (~66 um)	<5%
Triple-coil [99]	Al, Cu	Single frequency (200 kHz)	Up to 4	Thickness (0.4, 0.5 mm)	<3%

**Table 8:** Experimental evaluation of plate property estimation employing the representative coil inductance phenomenon.

Phenomenon	Plate Materials	Applied Frequency for Estimation	Lift-Off (mm)	Estimated Variables and Range	Relative Error of Estimation
Phase invariance phenomenon [143]	Al, Cu	100~1 MHz	-	Thickness (22 μm~5 mm)	<3%
Lift-off invariance inductance [24]	DP 600, DP 800	Single frequency depending on material	Up to 12	$\mu_{r}$	<1%
Lift-off invariance inductance [144]	DP 600, LC-Mild, Cr-Mo	Single frequency depending on material	Up to 5	$\sigma \;or\;\mu_{r}$	<1%
Conductivity invariance phenomenon [139]	DP 600, DP 800, DP 1000	Single frequency (90 kHz)	1.9	$\mu_{r}$	<3%
Permeability- independent frequency [141]	Al, Al alloy, SUS304	4~200 kHz	-	Thickness (1~8 mm)	-
Slope of lift-off curve [136]	Cu, Al, SS, Ti	1 MHz	0.04 to 0.4	Thickness (~100 um)	<3%

## Data Availability

Not applicable.

## References

[1] Xu J., Wu J., Xin W., Ge Z.L. (2020). Measuring Ultrathin Metallic Coating Properties Using Swept-Frequency Eddy-Current Technique. IEEE Trans. Instrum. Meas..

[2] Cheng W.Y. (2019). Swept-frequency eddy current testing to characterize a nonmagnetic metallic plate and a nonconductive coating over it. Int. J. Appl. Electromagn. Mech..

[3] Xu J., Wu J., Xin W., Ge Z.L. (2021). Fast Measurement of the Coating Thickness and Conductivity Using Eddy Currents and Plane Wave Approximation. IEEE Sens. J..

[4] AbdAlla A.N., Faraj M.A., Samsuri F., Rifai D., Ali K., Al-Douri Y. (2019). Challenges in improving the performance of eddy current testing: Review. Meas. Control.

[5] Hohmann R., Lomparski D., Krause H.J., Von Kreurzbruck M., Becker W. (2001). Aircraft wheel testing with remote eddy current technique using a HTS SQUID magnetometer. IEEE Trans. Appl. Supercond..

[6] Qi G.J., Lei H., Fu G.Q., Jing P., Lin J.M. (2012). In Situ Eddy-Current Testing on Low-Pressure Turbine Blades of Aircraft Engine. J. Test. Eval..

[7] Liu Z., Li W., Xue F.Q., Xiafang J.Y., Bu B., Yi Z. (2015). Electromagnetic Tomography Rail Defect Inspection. IEEE Trans. Magn..

[8] Augustyniak M., Borzyszkowski P., Bulawa M. (2019). Towards an Universal Method for Predicting Eddy-Current Sensor Characteristics in the Railway Industry. Nondestruct. Eval..

[9] Guilizzoni R., Finch G., Harmon S. (2019). Subsurface corrosion detection in industrial steel structures. IEEE Magn. Lett..

[10] Qu Z., Zhao Q., Meng Y. (2014). Improvement of sensitivity of eddy current sensors for nano-scale thickness measurement of Cu films. NDT E Int..

[11] Qu Z., Meng Y., Zhao Q. (2015). Eddy current measurement of the thickness of top Cu film of the multilayer interconnects in the integrated circuit (IC) manufacturing process. Front. Mech. Eng..

[12] Li H., Zhao Q., Lu X., Luo J. (2017). Signal processing and analysis for copper layer thickness measurement within a large variation range in the CMP process. Rev. Sci. Instrum..

[13] Dodd C.V., Luquire J., Deeds W., Spoeri W. (1969). Some Eddy-Current Problems and Their Integral Solutions.

[14] Dodd C., Deeds W. (1968). Analytical solutions to eddy-current probe-coil problems. J. Appl. Phys..

[15] Theodoulidis T., Kriezis E.E. (2006). Application of the TREE method to Axisymmetric Problems. Eddy Current Canonical Problems (with Applications to Nondestructive Evaluation).

[16] Huang R., Lu M., Zhang Z., Zhao Q., Xie Y., Tao Y., Meng T., Peyton A., Theodoulidis T., Yin W. (2020). Measurement of the radius of metallic plates based on a novel finite region eigenfunction expansion (FREE) method. IEEE Sens. J..

[17] Lu M., Yin L., Peyton A.J., Yin W. (2016). A novel compensation algorithm for thickness measurement immune to lift-off variations using eddy current method. IEEE Trans. Instrum. Meas..

[18] Zeng Z.W., Jiao S.N., Du F., Sun L., Li J. (2018). Eddy Current Testing of Delamination in Carbon Fiber Reinforced Polymer (CFRP): A Finite Element Analysis. Res. Nondestruct. Eval..

[19] Tian Q.Z., Zeng Z.W. (2020). Hybrid Formulation Domain-Decomposition Finite-Element Method for Simulating Eddy-Current Testing. IEEE Trans. Magn..

[20] Barbato L., Poulakis N., Tamburrino A., Theodoulidis T., Ventre S. (2015). Solution and Extension of a New Benchmark Problem for Eddy-Current Nondestructive Testing. IEEE Trans. Magn..

[21] Tesfalem H., Fletcher A.D., Brown M., Chapman B., Peyton A.J. (2018). Study of asymmetric gradiometer sensor configurations for eddy current based non-destructive testing in an industrial environment. NDT E Int..

[22] Huang R., Lu M., Chen Z., Shao Y., Xia Z., Hu G., Peyton A., Yin W. (2022). A novel acceleration method for crack computation using finite element analysis in eddy current testing. IEEE Trans. Instrum. Meas..

[23] Huang R., Lu M., Peyton A., Yin W. (2020). A novel perturbed matrix inversion based method for the acceleration of finite element analysis in crack-scanning eddy current NDT. IEEE Access.

[24] Lu M., Meng X., Huang R., Chen L., Peyton A., Yin W. (2020). Inversion of distance and magnetic permeability based on material-independent and liftoff insensitive algorithms using eddy current sensor. IEEE Trans. Instrum. Meas..

[25] Cui Z., Wang Q., Xue Q., Fan W., Zhang L., Cao Z., Sun B., Wang H., Yang W. (2016). A review on image reconstruction algorithms for electrical capacitance/resistance tomography. Sens. Rev..

[26] Lu M., Xie Y., Zhu W., Peyton A., Yin W. (2018). Determination of the magnetic permeability, electrical conductivity, and thickness of ferrite metallic plates using a multifrequency electromagnetic sensing system. IEEE Trans. Ind. Inform..

[27] Ali K.B., Abdalla A.N., Rifai D., Faraj M.A. (2017). Review on system development in eddy current testing and technique for defect classification and characterization. IET Circuits, Devices Syst..

[28] García-Martín J., Gómez-Gil J., Vázquez-Sánchez E. (2011). Non-destructive techniques based on eddy current testing. Sensors.

[29] Liu S., Sun Y., Jiang X., Kang Y. (2020). A review of wire rope detection methods, sensors and signal processing techniques. J. Nondestr. Eval..

[30] Sophian A., Tian G., Fan M. (2017). Pulsed eddy current non-destructive testing and evaluation: A review. Chin. J. Mech. Eng..

[31] Cheng W. (2021). Measurement of magnetic plates at a few hertz with two concentric coils and thickness estimation using mutual inductance. IEEE Trans. Instrum. Meas..

[32] Theodoulidis T.P., Bowler J.R. (2005). The Truncated Region Eigenfunction Expansion Method for the Solution of Boundary Value Problems in Eddy Current Nondestructive Evaluation. AIP Conf. Proc..

[33] Yin W., Peyton A.J., Dickinson S.J. (2004). Simultaneous measurement of distance and thickness of a thin metal plate with an electromagnetic sensor using a simplified model. IEEE Trans. Instrum. Meas..

[34] Huang R., Lu M., He X., Peyton A., Yin W. (2020). Measuring coaxial hole size of finite-size metallic disk based on a dual-constraint integration feature using multifrequency eddy current testing. IEEE Trans. Instrum. Meas..

[35] Huang R., Lu M., Peyton A., Yin W. (2020). Thickness measurement of metallic plates with finite planar dimension using eddy current method. IEEE Trans. Instrum. Meas..

[36] Pavo J., Miya K. (1994). Reconstruction of crack shape by optimization using eddy current field measurement. IEEE Trans. Magn..

[37] Yin W., Tang J., Lu M., Xu H., Huang R., Zhao Q., Zhang Z., Peyton A. (2019). An equivalent-effect phenomenon in eddy current non-destructive testing of thin structures. IEEE Access.

[38] Bowler J.R., Theodoulidis T.P., Poulakis N. (2012). Eddy current probe signals due to a crack at a right-angled corner. IEEE Trans. Magn..

[39] Du Y., Xie S., Li X., Chen Z., Uchimoto T., Takagi T. (2019). A fast forward simulation scheme for eddy current testing of crack in a structure of carbon fiber reinforced polymer laminate. IEEE Access.

[40] Zhao K., Vouvakis M.N., Lee J.-F. (2006). Solving electromagnetic problems using a novel symmetric FEM-BEM approach. IEEE Trans. Magn..

[41] Bíró O. (1999). Edge element formulations of eddy current problems. Comput. Methods Appl. Mech. Eng..

[42] Lu M., Peyton A., Yin W. (2017). Acceleration of frequency sweeping in eddy-current computation. IEEE Trans. Magn..

[43] Yin W., Lu M., Yin L., Zhao Q., Meng X., Zhang Z., Peyton A. (2018). Acceleration of eddy current computation for scanning probes. Insight Non-Destr. Test. Cond. Monit..

[44] Theodoulidis T. (2005). Analytical model for tilted coils in eddy-current nondestructive inspection. IEEE Trans. Magn..

[45] Chao X., Li Y., Nie J. (2016). Tilt angle measurement based on arrayed eddy current sensors. J. Magn..

[46] Burke S. (1990). Eddy-current induction in a uniaxially anisotropic plate. J. Appl. Phys..

[47] Beissner R., Sablik M. (1984). Theory of eddy currents induced by a nonsymmetric coil above a conducting half-space. J. Appl. Phys..

[48] Burke S., Ibrahim M. (2004). Mutual impedance of air-cored coils above a conducting plate. J. Phys. D Appl. Phys..

[49] Harfield N., Bowler J.R. (1997). Theory of thin-skin eddy-current interaction with surface cracks. J. Appl. Phys..

[50] Ditchburn R., Burke S., Posada M. (2003). Eddy-current nondestructive inspection with thin spiral coils: Long cracks in steel. J. Nondestruct. Eval..

[51] Lu M., Meng X., Huang R., Peyton A., Yin W. (2021). Analysis of tilt effect on notch depth profiling using thin-skin regime of driver-pickup eddy-current sensor. Sensors.

[52] O’Toole M.D., Karimian N., Peyton A.J. (2017). Classification of nonferrous metals using magnetic induction spectroscopy. IEEE Trans. Ind. Inform..

[53] Liu Y., Zhang Z., Yin W., Chen H., Yu Z., Wang Q. (2021). A Novel Conductivity Classification Technique for Nonmagnetic Metal Immune to Tilt Variations Using Eddy Current Testing. IEEE Access.

[54] Du Y., Zhang Z., Yin W., Zhu S., Chen Z., Xu H. (2020). Conductivity classification of non-magnetic tilting metals by eddy current sensors. Sensors.

[55] Du Y., Zhang Z., Yin W., Tytko G. (2021). Sloping-invariance for nonferrous metallic slabs at multiple frequencies by eddy current sensors. IEEE Access.

[56] Hampton J., Fletcher A., Tesfalem H., Peyton A., Brown M. (2022). A comparison of non-linear optimisation algorithms for recovering the conductivity depth profile of an electrically conductive block using eddy current inspection. NDT E Int..

[57] Uzal E., Moulder J., Rose J. (1994). Experimental determination of the near-surface conductivity profiles of metals from electromagnetic induction (eddy current) measurements. Inverse Probl..

[58] Cai W., Jomdecha C., Zhao Y., Wang L., Xie S., Chen Z. (2020). Quantitative evaluation of electrical conductivity inside stress corrosion crack with electromagnetic NDE methods. Philos. Trans. R. Soc. London. Ser. A.

[59] Ge J., Yusa N., Fan M. (2021). Frequency component mixing of pulsed or multi-frequency eddy current testing for nonferromagnetic plate thickness measurement using a multi-gene genetic programming algorithm. NDT E Int..

[60] Meng X., Lu M., Yin W., Bennecer A., Kirk K.J. (2020). Inversion of lift-off distance and thickness for nonmagnetic metal using eddy current testing. IEEE Trans. Instrum. Meas..

[61] Chen X., Li J., Wang Z. (2020). Inversion method in pulsed eddy current testing for wall thickness of ferromagnetic pipes. IEEE Trans. Instrum. Meas..

[62] Haddar H., Jiang Z., Riahi M.K. (2017). A robust inversion method for quantitative 3D shape reconstruction from coaxial eddy current measurements. J. Sci. Comput..

[63] Du Y., Li X., Xie S., Yang S., Chen Z. (2020). Reconstruction of cracks in a carbon fiber-reinforced polymer laminate plate from signals of eddy current testing. J. Compos. Mater..

[64] Adewale I.D., Tian G.Y. (2012). Decoupling the influence of permeability and conductivity in pulsed eddy-current measurements. IEEE Trans. Magn..

[65] Vasic D., Bilas V., Ambrus D. (2006). Validation of a coil impedance model for simultaneous measurement of electromagnetic properties and inner diameter of a conductive tube. IEEE Trans. Instrum. Meas..

[66] Vasić D., Bilas V. Lumped representation in inductive measurement of metal casing properties. In Proceedings of 2010 IEEE Instrumentation & Measurement Technology Conference Proceedings.

[67] Ghaffari A., Krstić M., Nešić D. (2012). Multivariable Newton-based extremum seeking. Automatica.

[68] Schöbi R. (2019). Surrogate models for uncertainty quantification in the context of imprecise probability modelling. IBK Ber..

[69] Zhang J., Yin J., Wang R. (2020). Basic framework and main methods of uncertainty quantification. Math. Probl. Eng..

[70] Aster R.C., Borchers B., Thurber C.H., Aster R.C., Borchers B., Thurber C.H. (2019). Chapter Three—Rank Deficiency and Ill-Conditioning. Parameter Estimation and Inverse Problems.

[71] Salcedo-Sanz S. (2016). Modern meta-heuristics based on nonlinear physics processes: A review of models and design procedures. Phys. Rep..

[72] Parouha R.P., Verma P. (2021). State-of-the-art reviews of meta-heuristic algorithms with their novel proposal for unconstrained optimization and applications. Arch. Comput. Methods Eng..

[73] Lu M. (2018). Forward and Inverse Analysis for non-Destructive Testing Based on Electromagnetic Computation Methods. Ph.D. Thesis.

[74] Hampton J., Tesfalem H., Dorn O., Fletcher A., Peyton A., Brown M. (2022). Calibration of a Finite Element Forward Model in Eddy Current Inspection. IEEE Sens. J..

[75] Wu J., Zhou D., Wang J., Guo X., You L., An W., Zhang H. Surface crack detection for carbon fiber reinforced plastic (CFRP) materials using pulsed eddy current testing. In Proceedings of 2014 IEEE Far East Forum on Nondestructive Evaluation/Testing.

[76] O’Toole M.D., Karimian N., Peyton A.J. Fast classification of non-magnetic metal targets using eddy-current based impedance spectroscopy. In Proceedings of 2017 IEEE SENSORS.

[77] Tao Y., Xu H., Chen Z., Huang R., Yin W. Automatic feature extraction method for crack detection in eddy current testing. In Proceedings of 2019 IEEE International Instrumentation and Measurement Technology Conference (I2MTC).

[78] Tao Y., Ktistis C., Zhao Y., Yin W., Peyton A.J. (2019). A Class D Power Amplifier for Multifrequency Eddy Current Testing Based on Multisimultaneous-Frequency Selective Harmonic Elimination Pulsewidth Modulation. IEEE Trans. Ind. Electron..

[79] Bernieri A., Betta G., Ferrigno L., Laracca M. Multi-frequency ECT method for defect depth estimation. In Proceedings of IEEE Sensors Applications Symposium.

[80] Yin W., Dickinson S.J., Peyton A.J. (2006). A multi-frequency impedance analysing instrument for eddy current testing. Meas. Sci. Technol..

[81] Avila J., Chen Z., Xu H., Yin W. A multi-frequency NDT system for imaging and detection of cracks. In Proceedings of 2018 IEEE International Symposium on Circuits and Systems (ISCAS).

[82] Betta G., Ferrigno L., Laracca M., Burrascano P., Ricci M. Optimized complex signals for eddy current testing. In Proceedings of 2014 IEEE International Instrumentation and Measurement Technology Conference (I2MTC) Proceedings.

[83] Yin W., Chen G., Chen L., Wang B. (2011). The Design of a Digital Magnetic Induction Tomography (MIT) System for Metallic Object Imaging Based on Half Cycle Demodulation. IEEE Sens. J..

[84] Yin W., Chen G., Jian J., Cui Z. The design of a FPGA-based digital magnetic induction tomography (MIT) system for metallic object imaging. In Proceedings of IEEE Instrumentation & Measurement Technology Conference.

[85] Wei H.Y., Soleimani M. (2012). Hardware and software design for a National Instrument-based magnetic induction tomography system for prospective biomedical applications. Physiol. Meas..

[86] Hanyang X., Avila S., Ricardo J., Fanfu W., Roy M.J., Xie Y., Zhou F. (2018). Imaging x70 weld cross-section using electromagnetic testing. NDT E Int..

[87] Zhu W., Yang H., Luinenburg A., Van Den Berg F., Dickinson S., Yin W., Peyton A.J. (2014). Development and deployment of online multifrequency electromagnetic system to monitor steel hot transformation on runout table of hot strip mill. Ironmak. Steelmak..

[88] Yin W., Peyton A.J. (2006). A planar EMT system for the detection of faults on thin metallic plates. Meas. Sci. Technol..

[89] Chao W., He H., Cui Z., Cao Q., Ping Z., Wang H. (2018). A novel EMT system based on TMR sensors for reconstruction of permeability distribution. Meas. Sci. Technol..

[90] Ma X., Peyton A.J., Higson S.R., Lyons A., Dickinson S.J. (2006). Hardware and software design for an electromagnetic induction tomography (EMT) system for high contrast metal process applications. Meas. Sci. Technol..

[91] Kekelj M., Bulic N., Sucic V. An FPGA implementation of the Goertzel algorithm in a Non-Destructive Eddy current Testing. In Proceedings of 2017 International Conference on Signals and Systems (ICSigSys).

[92] Hamel M., Mohellebi H. (2020). A LabVIEW-based real-time acquisition system for crack detection in conductive materials. Math. Comput. Simul..

[93] Zhang N., Peng L., He Y., Ye C. (2022). Flexible Probe With Array Tunneling Magnetoresistance Sensors for Curved Structure Inspection. IEEE Trans. Instrum. Meas..

[94] Soni A.K., Rao B.P. (2018). Lock-in Amplifier Based Eddy Current Instrument for Detection of Sub-surface Defect in Stainless Steel Plates. Sens. Imaging.

[95] Aguiam D.E., Rosado L.S., Ramos P.M., Piedade M. (2015). Heterodyning based portable instrument for eddy currents non-destructive testing. Measurement.

[96] Zhang G., Xie X., You Y. (2021). Multi-Channel Eddy Current Detector Based on Virtual Instrument Technology and Self-Balancing Technology. Sens. Imaging.

[97] Dingley G., Soleimani M. (2021). Multi-Frequency Magnetic Induction Tomography System and Algorithm for Imaging Metallic Objects. Sensors.

[98] Technologies Keysight Impedance Measurement Handbook. www.keysight.com.

[99] Lu M., Meng X., Yin W., Qu Z., Wu F., Tang J., Xu H., Huang R., Chen Z., Zhao Q. (2019). Thickness measurement of non-magnetic steel plates using a novel planar triple-coil sensor. NDT E Int..

[100] Yin W., Xu K. (2015). A novel triple-coil electromagnetic sensor for thickness measurement immune to lift-off variations. IEEE Trans. Instrum. Meas..

[101] Rosell-Ferrer J., Merwa R., Brunner P., Scharfetter H. (2006). A multifrequency magnetic induction tomography system using planar gradiometers: Data collection and calibration. Physiol. Meas..

[102] Abdel-Rehim O.A., Davidson J.L., Marsh L.A., O’Toole M.D., Peyton A.J. (2016). Magnetic Polarizability Tensor Spectroscopy for Low Metal Anti-personnel Mine Surrogates. IEEE Sens. J..

[103] Chen Z., Salas-Avlia J.R., Tao Y., Yin W., Ricardo J. (2020). A novel hybrid serial/parallel multi-frequency measurement method for impedance analysis in eddy current testing. Rev. Sci. Instrum..

[104] Xu Z., Wang X., Deng Y. (2020). Rotating focused field Eddy-current sensing for arbitrary orientation defects detection in carbon steel. Sensors.

[105] Center for Nondestructive Evaluation Nondestructive Evaluation Techniques. https://www.nde-ed.org/NDETechniques/EddyCurrent/ProbesCoilDesign/ProbesConfig.xhtml.

[106] Gao P., Wang C., Li Y., Cong Z. (2015). Electromagnetic and eddy current NDT in weld inspection: A review. Insight Non-Destruct. Test. Cond. Monit..

[107] Sharif N.A., Ramli R., Nuawi M.Z., Mohamed A.Z. (2020). Theory and development of magnetic flux leakage sensor for flaws detection: A review. J. Kejuruter..

[108] Halchenko V., Trembovetskaya R., Tychkov V. (2020). Surface Eddy Current Probes: Excitation Systems of the Optimal Electromagnetic Field (Review). Devices Methods Meas..

[109] Hoshikawa H., Koyama K. (1999). Eddy current distribution using parameters normalized by standard penetration depth. Review of Progress in Quantitative Nondestructive Evaluation.

[110] Mook G., Hesse O., Uchanin V. (2007). Deep penetrating eddy currents and probes. Mater. Test..

[111] Jiao S., Liu X., Zeng Z. (2017). Intensive study of skin effect in eddy current testing with pancake coil. IEEE Trans. Magn..

[112] Zeng Z., Ding P., Li J., Jiao S., Lin J., Dai Y. (2019). Characteristics of Eddy Current Attenuation and Thickness Measurement of Metallic Plate. Chin. J. Mech. Eng..

[113] Hayashi M., Saito T., Nakamura Y., Sakai K., Kiwa T., Tanikura I., Tsukada K. (2019). Extraction Method of Crack Signal for Inspection of Complicated Steel Structures Using A Dual-Channel Magnetic Sensor. Sensors.

[114] Yin W., Peyton A.J. (2007). Thickness measurement of non-magnetic plates using multi-frequency eddy current sensors. NDT E Int..

[115] Mori H., Kagaya H., Inamoto Y., Izumi S.I., Yashima K., Takagi T. (2020). Numerical Analysis of Eddy Current Distribution in Submental Region Induced by Magnetic Stimulation for Treating Dysphagia. IEEE Trans. Neural Syst. Rehabil. Eng..

[116] Chen D., Li Y., Pan M., Tian W. (2018). Flexible planar electromagnetic sensor array fabricated with printing electronic technology. Measurement.

[117] Daura L.U., Tian G., Yi Q., Sophian A. (2020). Wireless power transfer-based eddy current non-destructive testing using a flexible printed coil array. Philos. Trans. R. Soc. London. Ser. A.

[118] Avila J.R.S., How K.Y., Lu M., Yin W. (2017). A novel dual modality sensor with sensitivities to permittivity, conductivity, and permeability. IEEE Sens. J..

[119] Hamia R., Cordier C., Dolabdjian C. (2014). Eddy-current non-destructive testing system for the determination of crack orientation. NDT E Int..

[120] Dogaru T., Smith C.H., Schneider R.W., Smith S.T. (2004). Deep crack detection around fastener holes in airplane multi-layered structures using GMR-based eddy current probes. AIP Conf. Proc..

[121] Ye C., Huang Y., Udpa L., Udpa S.S. (2015). Differential sensor measurement with rotating current excitation for evaluating multilayer structures. IEEE Sens. J..

[122] Ye C., Rosell A., Haq M., Stitt E., Udpa L., Udpa S. (2019). EC probe with orthogonal excitation coils and TMR sensor for CFRP inspection. Int. J. Appl. Electromagn. Mech..

[123] Ripka P., Janosek M. (2010). Advances in magnetic field sensors. IEEE Sens. J..

[124] Hainz S., de la Torre E., Güttinger J. Comparison of magnetic field sensor technologies for the use in wheel speed sensors. In Proceedings of 2019 IEEE International Conference on Industrial Technology (ICIT).

[125] Hadjigeorgiou N., Asimakopoulos K., Papafotis K., Sotiriadis P.P. (2020). Vector Magnetic Field Sensors: Operating Principles, Calibration, and Applications. IEEE Sens. J..

[126] Lu M., Meng X., Huang R., Chen L., Peyton A., Yin W., Qu Z. (2021). Thickness measurement of circular metallic film using single-frequency eddy current sensor. NDT E Int..

[127] Lu M., Meng X., Huang R., Chen L., Peyton A., Yin W. (2021). A high-frequency phase feature for the measurement of magnetic permeability using eddy current sensor. NDT E Int..

[128] Wang C., Wang R.C., Liang X., Ye J.M., Chen X.Y. (2022). Design and optimization of electromagnetic tomography and electrical resistance tomography dual-modality sensor. Meas. Sci. Technol..

[129] Arellano Y., Hunt A., Haas O., Ahmed H., Ma L. (2019). Multiple regression-based prediction correlations for enhanced sensor design of magnetic induction tomography systems. Meas. Sci. Technol..

[130] She S., Chen Y., He Y., Zou X. (2021). Optimal design of remote field eddy current testing probe for ferromagnetic pipeline inspection. Measurement.

[131] Avila J.R.S., Lu M., Huang R., Chen Z., Zhu S., Yin W. (2020). Accurate measurements of plate thickness with variable lift-off using a combined inductive and capacitive sensor. NDT E Int..

[132] Lu M., Huang R., Yin W., Zhao Q., Peyton A. (2019). Measurement of permeability for ferrous metallic plates using a novel lift-off compensation technique on phase signature. IEEE Sens. J..

[133] Lu M., Meng X., Huang R., Chen L., Peyton A., Yin W. (2020). Measuring lift-off distance and electromagnetic property of metal using dual-frequency linearity feature. IEEE Trans. Instrum. Meas..

[134] Dinh C.-H., Jeng J.-T., Huang G.-W., Chen J.-Y., Chiang Y.-S., Doan V.-D., Pham T.-T. (2021). Real-time thickness measurement using resonant eddy-current sensor. IEEE Trans. Instrum. Meas..

[135] Lu M., Zhu W., Yin L., Peyton A.J., Yin W., Qu Z. (2017). Reducing the lift-off effect on permeability measurement for magnetic plates from multifrequency induction data. IEEE Trans. Instrum. Meas..

[136] Wang H., Li W., Feng Z. (2015). Noncontact thickness measurement of metal films using eddy-current sensors immune to distance variation. IEEE Trans. Instrum. Meas..

[137] Li W., Wang H., Feng Z. (2016). Non-contact online thickness measurement system for metal films based on eddy current sensing with distance tracking technique. Review of Scientific Instrum..

[138] Yin W., Dickinson S.J., Peyton A. (2005). Imaging the continuous conductivity profile within layered metal structures using inductance spectroscopy. IEEE Sens. J..

[139] Lu M., Xu H., Zhu W., Yin L., Zhao Q., Peyton A., Yin W. (2018). Conductivity Lift-off Invariance and measurement of permeability for ferrite metallic plates. NDT E Int..

[140] Yin W., Meng X., Lu M., Zhao Q., Xu H., Zhang Z., Peyton A. (2019). Permeability invariance phenomenon and measurement of electrical conductivity for ferrite metallic plates. Insight—Non-Destruct. Test. Cond. Monit..

[141] Cheng W. (2017). Thickness measurement of metal plates using swept-frequency eddy current testing and impedance normalization. IEEE Sens. J..

[142] Yin W., Peyton A.J. (2008). Thickness measurement of metallic plates with an electromagnetic sensor using phase signature analysis. IEEE Trans. Instrum. Meas..

[143] Angani C.S., Ramos H.G., Ribeiro A.L., Rocha T.J., Baskaran P. (2016). Lift-off point of intersection feature in transient eddy-current oscillations method to detect thickness variation in stainless steel. IEEE Trans. Magn..

[144] Lu M., Meng X., Huang R., Chen L., Peyton A., Yin W. (2021). Lift-off invariant inductance of steels in multi-frequency eddy-current testing. NDT E Int..

[145] Mohseni E., Habibzadeh Boukani H., Ramos França D., Viens M. (2020). A study of the automated eddy current detection of cracks in steel plates. J. Nondestruct. Eval..

[146] Li W., Chen G., Ge J., Yin X., Li K. (2016). High sensitivity rotating alternating current field measurement for arbitrary-angle underwater cracks. NDT E Int..

[147] Pasadas D.J., Ribeiro A.L., Ramos H.G., Rocha T.J. (2017). Inspection of cracks in aluminum multilayer structures using planar ECT probe and inversion problem. IEEE Trans. Instrum. Meas..

[148] Yin L., Ye B., Zhang Z., Tao Y., Xu H., Avila J.R.S., Yin W. (2019). A novel feature extraction method of eddy current testing for defect detection based on machine learning. NDT E Int..

[149] Bajracharya S., Sasaki E., Tamura H. (2019). Numerical study on corrosion profile estimation of a corroded steel plate using eddy current. Struct. Infrastruct. Eng..

[150] Xu H., Lu M., Avila J., Zhao Q., Zhou F., Meng X., Yin W. (2019). Imaging a weld cross-section using a novel frequency feature in multi-frequency eddy current testing. Insight—Non-Destructive Test. Cond. Monit..

[151] Ribeiro A.L., Alegria F., Postolache O.A., Ramos H.M.G. (2010). Liftoff correction based on the spatial spectral behavior of eddy-current images. IEEE Trans. Instrum. Meas..

[152] Shao Y., Meng T., Yu K., Xia Z., Huang R., Tao Y., Chen Z., Avila J.R.S., Yin W. (2022). Automatic Detection and Imaging of Rivet Hole Defects for Aircraft Structures with Optimised Sensor Array Using Eddy Current Method and Image Analysis. IEEE Sens. J..

[153] Ferrigno L., Laracca M., Malekmohammadi H., Tian G.Y., Ricci M. (2019). Comparison of time and frequency domain features’ immunity against lift-off in pulse-compression eddy current imaging. NDT E Int..

[154] Betta G., Ferrigno L., Laracca M., Ramos H., Ricci M., Ribeiro A., Silipigni G. Fast 2D crack profile reconstruction by image processing for Eddy-Current Testing. In Proceedings of 2015 IEEE Metrology for Aerospace (MetroAeroSpace).

[155] Wang Y., Chen Y., Zhang Y., Chen H., Yu S. (2016). Generalised Hermite–Gaussian beams and mode transformations. J. Opt..

[156] Ahmed S., Reboud C., Lhuillier P.-E., Calmon P., Miorelli R. (2019). An adaptive sampling strategy for quasi real time crack characterization on eddy current testing signals. NDT E Int..

[157] Fan M., Wang Q., Cao B., Ye B., Sunny A.I., Tian G. (2016). Frequency optimization for enhancement of surface defect classification using the eddy current technique. Sensors.

[158] Rosado L.S., Janeiro F.M., Ramos P.M., Piedade M. (2013). Defect characterization with eddy current testing using nonlinear-regression feature extraction and artificial neural networks. IEEE Trans. Instrum. Meas..

[159] Deng W., Bao J., Ye B. (2020). Defect Image Recognition and Classification for Eddy Current Testing of Titanium Plate Based on Convolutional Neural Network. Complexity.

[160] Meng T., Tao Y., Chen Z., Avila J.R.S., Ran Q., Shao Y., Huang R., Xie Y., Zhao Q., Zhang Z. (2021). Depth evaluation for metal surface defects by eddy current testing using deep residual convolutional neural networks. IEEE Trans. Instrum. Meas..

[161] Hampe N., Katscher U., Van den Berg C.A., Tha K.K., Mandija S. (2020). Investigating the challenges and generalizability of deep learning brain conductivity mapping. Phys. Med. Biol..

